# Age-Specific Physiological Adjustments of *Spirodela polyrhiza* to Sulfur Deficiency

**DOI:** 10.3390/plants14131907

**Published:** 2025-06-20

**Authors:** Vesna Peršić, Anja Melnjak, Lucija Domjan, Günther Zellnig, Jasenka Antunović Dunić

**Affiliations:** 1 Department of Biology, Josip Juraj Strossmayer University of Osijek, 31000 Osijek, Croatia; vpersic@biologija.unios.hr (V.P.); anja.melnjak@biologija.unios.hr (A.M.); lucija.domjan96@gmail.com (L.D.); 2 Institute of Biology, University of Graz, 8010 Graz, Austria

**Keywords:** chloroplast ultrastructure, anthocyanins, starch accumulation, oxygen evolution rates, chlorophyll *a* fluorescence kinetics, rapid light curve, MR_820_

## Abstract

*Spirodela polyrhiza* is a suitable model organism for investigating plant developmental influences due to its intracolonial variations in response to various environmental fluctuations, like nutrient deficiency. In this study, transmission electron microscopy was used to examine age-dependent variation in chloroplast ultrastructure, while pigment levels (chlorophyll and anthocyanins), starch accumulation, and metabolic activity (photosynthetic and respiratory rates) were measured to determine metabolic responses to sulfur deficiency. For a comprehensive insight into electron transport efficiency and the redox states of the photosynthetic apparatus, rapid light curves, chlorophyll fluorescence (JIP test parameters), and modulated reflection at 820 nm were analyzed. Under S deficit, mother fronds relied on stored reserves to maintain functional PSII but accumulated reduced PQ pools, slowing electron flow beyond PSII. The first-generation daughter fronds, despite having higher baseline photosynthetic capacity, exhibited the largest decline in photosynthetic indicators (e.g., rETR fell about 50%), limitations in the water-splitting complex, and reduced PSI end-acceptor capacity that resulted in donor- and acceptor-side bottlenecks of electron transport. The youngest granddaughter fronds avoided these bottlenecks by absorbing less light per PSII, channeling electrons through the alternative pathway to balance PQ pools and redox-stable PSI while diverting more carbon into starch and anthocyanin production up to 5-fold for both. These coordinated and age-specific adjustments that provide response flexibility may help maintain photosynthetic function of the colony and facilitate rapid recovery when sulfur becomes available again.

## 1. Introduction

Plants optimize the use of available sulfur (S) to meet the demands for growth, development, and stress resistance [1]. Sulfur is one of the essential macronutrients, and, as a structural component of protein disulfide bonds, amino acids, enzymes, cofactors, and vitamins, its deficiency reduces plant metabolic activity [2,3]. The physiological and biochemical effects of sulfur starvation have been studied in various algae and plant species. For instance, due to sulfur deficiency, the sequential accumulation of starch and lipids commonly occurs in algae [4,5] and duckweeds [6]. Long-term sulfur deprivation shows that sulfur deficiency modifies energy and redox status in plant tissue, severely decreasing ATP levels, but modifications in respiratory chain activity and mitochondrial ultrastructure lead to a new homeostasis, ensuring survival [7].

Furthermore, it has been determined that sulfur starvation in *Lemna minor* leads to preferential degradation of RuBP carboxylase/oxygenase (Rubisco) in a process that does not lead to plant death, implying that Rubisco is a sulfur storage protein [8]. Similarly, under sulfur deficiency, Rubisco degradation was faster than the total soluble protein in C3 plants like *L. minor* and wheat, while the opposite was observed in maize and sorghum, C4 plants [9]. In addition, sulfate deficiency in rice reduced the CO_2_ assimilation rate by inhibiting the synthesis of Rubisco, creating a traffic jam of electrons that over-reduces the plastoquinone pool, thereby restricting primary energy production [10].

Most investigations dealing with the effect of S deficiency have been conducted either at a specific developmental stage of the plant or are focused on mature plants. Few, such as Gilbert et al. [11], examine the relationship between Rubisco activity in wheat leaves of different developmental stages during S deprivation, in which they show reduced Rubisco activity and CO_2_ assimilation rates due to limitations in the de novo synthesis of photosynthetic components in younger leaves, but, as S deprivation prolongs, photosynthetic efficiency decreases in all leaves. Likewise, Aarabi et al. [12] used time-course sampling during seed filling in Arabidopsis to demonstrate that sulfur deficiency accelerates the synthesis of storage proteins, redirecting the plants from the growth phase to the maturation phase earlier and explaining a regulatory mechanism that, depending on the sulfur levels and developmental stage, activates or deactivates the SDI1 and SDI2 genes, and thus adjusts the growth strategy.

The primary subject of our research was the floating aquatic plant *Spirodela polyrhiza*, also commonly referred to as great or giant duckweed. This small and simple monocot is easily cultivated and managed in both laboratory and outdoor settings, and its rapid growth enables it to double its biomass in 2–3 days under optimal conditions, or in 3–5 days in the environment [13,14]. In addition, *S. polyrhiza*, with its small genome (158 Mb), coupled with extremely low genetic variation and spontaneous mutation rate [15,16], has become an ideal model organism for real-time monitoring processes in experimental evolutionary and functional studies [17,18]. Its morphology resembles the juvenile stages of more complex plants, and this neotenous reduction is thought to be an adaptation to the aquatic habitat [15], but with a highly efficient photosynthetic mechanism, enabling dynamic adjustment to environmental challenges [6]. Reduced complexity reduces energy consumption for creating and maintaining complex structures, making them highly competitive in their niche but also very convenient for studying physiological responses.

According to Harkess et al. [19], *S. polyrhiza* has high levels of proteins linked to chloroplast function, indicating their high optimization level for generating energy through photosynthesis. Additionally, evidence from a related duckweed, *Lemna trisulca*, suggests that its chloroplasts can move when exposed to heavy metals, implying that such mobility may help duckweeds quickly adapt by reorganizing their internal structures [20]. Thus, chloroplast dynamics and high optimization of photosynthesis enable duckweeds to rapidly reallocate energy and adjust their metabolism in response to a fluctuating environment [21]. Because *S. polyrhiza* exhibits rapid growth, stress-induced adjustments of photosynthesis can be monitored almost in real time. The literature shows that *S. polyrhiza* dynamically reconfigures its photosynthetic apparatus in response to changes in the environmental conditions through coordinated structural [21] and physiological [5] adaptations. Heavy metals oxidize PSII core D1 protein and induce photochemical inactivation [22], while salt stress downregulates transcripts for electron carriers and state transition regulators of light reactions [23]. Heat stress induces chloroplast transcriptional reprogramming to stabilize thylakoid membranes and maintain grana stacking [24], while under nutrient stress, *S. polyrhiza* suppresses energy-conserving genes to support survival strategy under deprivation [25].

These adjustments may also be important during, for example, nutrient deficiencies, as the photosynthetic machinery may need to adapt differently depending on the frond’s stage of development by ensuring that at least some offspring survive [26]. For example, younger fronds might have a different capacity for coping with stress than older ones, leading to age-specific differences in photosynthetic efficiency and overall growth. It was determined previously that frond age significantly influences the resilience and functional stability of photosynthesis in *S. polyrhiza* when exposed to cadmium toxicity [22]. Similarly, Oláh et al. [18] highlighted the importance of considering ontogenetic differences in ecotoxicological and physiological studies of duckweeds. They determined that age significantly influences sensitivity and adaptation patterns in duckweed fronds, and that even within frond spatial distribution of stress effects varies significantly among duckweed species and tested metals. Thus, the complexity of plant responses to environmental stress underscores the importance of considering not just species-specific but also ontogenetic contexts in ecotoxicological assessments.

This research aimed to investigate how the developmental stage modulates physiological, metabolic, and photosynthetic responses in *S. polyrhiza* under sulfur-deficiency conditions. Furthermore, this is the first study to connect chloroplast ultrastructure, rapid light curves, JIP test kinetics, and modulated reflection at 820 nm in higher plants under sulfur-deficient conditions, providing a multi-scale view of electron transport bottlenecks. Based on previous research, we hypothesized differential sensitivity and adaptive responses among fronds of different ages in the colony and presume greater metabolic plasticity in younger than older fronds sustained by ultrastructural adjustments, carbon re-allocation, and activation of alternative electron sinks, all to conserve photosynthetic activity. Therefore, this study provides the first quantitative comparison of fronds of different ages within the same *S. polyrhiza* colony, revealing an intracolonial distribution of response to nutrient stress. This intracolonial variation in response is important for understanding how developmental stages influence plant resilience and adaptation, as well as how these age-dependent responses contribute to duckweeds’ overall fitness and adaptability in fluctuating environments.

## 2. Results

### 2.1. Age-Dependent Variation in Chloroplast Ultrastructure of S. polyrhiza

**Figure 1:**
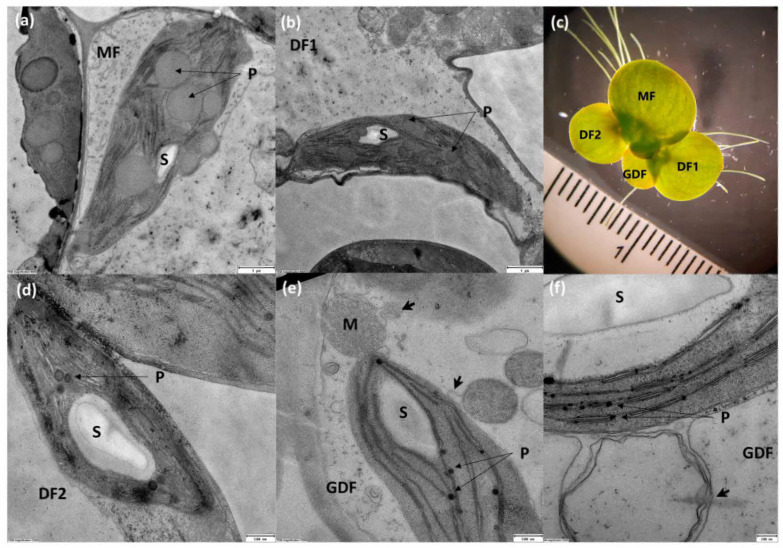
*S. polyrhiza* four-frond colony (**c**) with micrographs of chloroplasts in optimal control conditions: (**a**) chloroplasts in mother fronds (MF), (**b**) first daughter fronds (DF1), (**d**) second daughter fronds (DF2), (**e**) granddaughter fronds (GDF); (**f**) closer look at the thylakoids of the youngest granddaughter frond. S—starch grain, P—plastoglobuli, M—mitochondria, arrowheads represent tubular protrusions on mitochondria and the tonoplast (**f**), which surrounds another membrane (of unclear origin).

Transmission electron microscopy (TEM) revealed that chloroplasts in *S. polyrhiza* have the same general chloroplast architecture, double-membrane envelopes, stacked (grana) thylakoids, stromal thylakoids, starch granules (S), and plastoglobuli (P) across all fronds of different age in a four-frond colony in control optimal conditions (Figure 1). There are some differences regarding the thylakoid structure and the size and abundance of starch grains and plastoglobuli as fronds age. Mother fronds (MF) are the oldest fronds, characterized by well-developed grana thylakoids in their chloroplasts, smaller starch grains, and relatively abundant large plastoglobuli (Figure 1a). Chloroplasts in the first daughter frond (the oldest daughter, DF1) were similar in size and structure, with starch grains, stacked grana thylakoids connected to stromal thylakoids, and fewer, smaller plastoglobuli than in MF (Figure 1b). In the younger daughter frond (DF2), the structure of the chloroplast is similar to DF1 but with a larger starch grain. Granum thylakoids are interconnected with stromal thylakoids, with plastoglobuli present in smaller numbers and sizes than in the older daughter frond (Figure 1d). The youngest granddaughter frond (GDF) has slightly different chloroplasts in structure and size than older fronds, with protrusions on chloroplasts and mitochondria. Thylakoids have fewer distinctively visible grana stacks connected with stromal lamellae. Larger starch grains are present, and plastoglobuli are small and associated with thylakoids (Figure 1e,f). Since chloroplasts and their starch granules are anisotropic and light-oriented, a 2D profile can be biased in providing a reliable morphometry, especially because the tissue was embedded without prior randomization, and sections were not cut under an isotropic–uniform–random protocol. However, since plastoglobuli are nearly spherical and their visible cross-section does not depend on orientation, the elementary geometry of plastoglobuli in all experimental groups is presented in Table 1. Kruskal-Wallis ANOVA indicated a significant effect of *S. polyrhiza* frond age on plastoglobuli diameter (H(3) = 85.83, *p* < 0.0001). Dunn-Bonferroni post-hoc tests showed an increase in plastoglobuli size with age (MF > DF1 > DF2 > GDF), with all pairwise differences significant except DF1–DF2 and DF2–GDF (Table 1).

### 2.2. Pigments, Total Anthocyanins, and Starch

**Figure 2:**
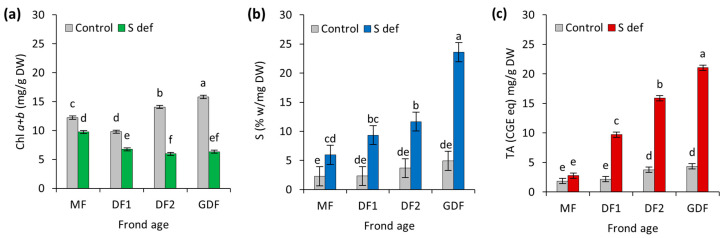
Chlorophyll *a*+*b* content (mg/g DW) (**a**), starch content (S, % w/mg DW) (**b**), and total anthocyanins (TA in CGE eq mg/g DW) (**c**) in *Spirodela polyrhiza* fronds of different age, under control and sulfur-deficient (S def) conditions. Frond age categories include mother fronds (MF, the oldest), first daughter fronds (DF1), second daughter fronds (DF2), and granddaughter fronds (GDF, the youngest). Bars represent mean values with 95% confidence intervals (n = 6). Different letters indicate statistically significant differences between means (Tukey HSD, *p* < 0.05).

Frond age (FA) and sulfur deficiency significantly reduced the chlorophyll *a+b* content in *S. polyrhiza* (two-way ANOVA F_7,40_ = 716.8, *p* < 0.0001), with S-deficit as the most influential variable, exhibiting a very large standardized effect size (Cohen’s f = 9.36, results of all further statistical data analyses are provided in Supplementary Materials, Table S1). Frond age also contributed to the variation in chlorophyll content with a lesser effect, indicating that the reduction in chlorophyll content was not uniform across all frond ages. Under S-deficit, chlorophyll content was reduced by 20–30% in the older mother fronds (MF and DF1) and by up to 58% and 60% in the younger DF2 and GDF, respectively (Figure 2a).

On the other hand, sulfur-deficient fronds showed a significant increase (*p* < 0.0001) in starch content compared to the control across all ages (Table S1, Figure 2b). In MF, starch content increased 2-fold under S-deficit. The increase in DF1 and DF2 was approximately 3-fold, while the most significant increase was in the youngest fronds (GDF), where starch content was 5-fold higher than in the control GDF (or 379%). Statistical analysis showed that S deficiency contributed nearly half of the variance observed in starch content, while FA was less dominant, explaining one-third of the variance, and, despite interaction being significant, it accounted for a smaller portion of the variance (Table S1). Therefore, the differences in starch accumulation under S-deficit depended on the frond age, making S-deficit a primary driver of starch accumulation. Although FA showed a similar magnitude of difference, the effect was localized only to the deficient subgroup.

A similar effect was observed in total anthocyanin content. Sulfur deficiency strongly induced anthocyanin accumulation (*p* < 0.0001), and the increase in anthocyanins depended on the FA, with younger fronds exhibiting higher accumulation. Similar to starch accumulation, the S-deficit explained nearly 50% of the total variance with a very large effect size (Table S1), followed by the frond age and their interaction, suggesting that younger fronds respond strongly to sulfur deficiency. In the oldest fronds (MF), anthocyanin content did not change under S-deficit, while the increase in daughter fronds DF1, DF2, and GDF was 4.5-fold, 4.2-fold, and 4.8-fold, respectively (Figure 2c).

These results indicate that younger fronds were more responsive to S-deficit, with a larger decrease in pigment content and a greater increase in starch and total anthocyanins.

### 2.3. Net Photosynthesis and Dark Respiration Rates

**Figure 3:**
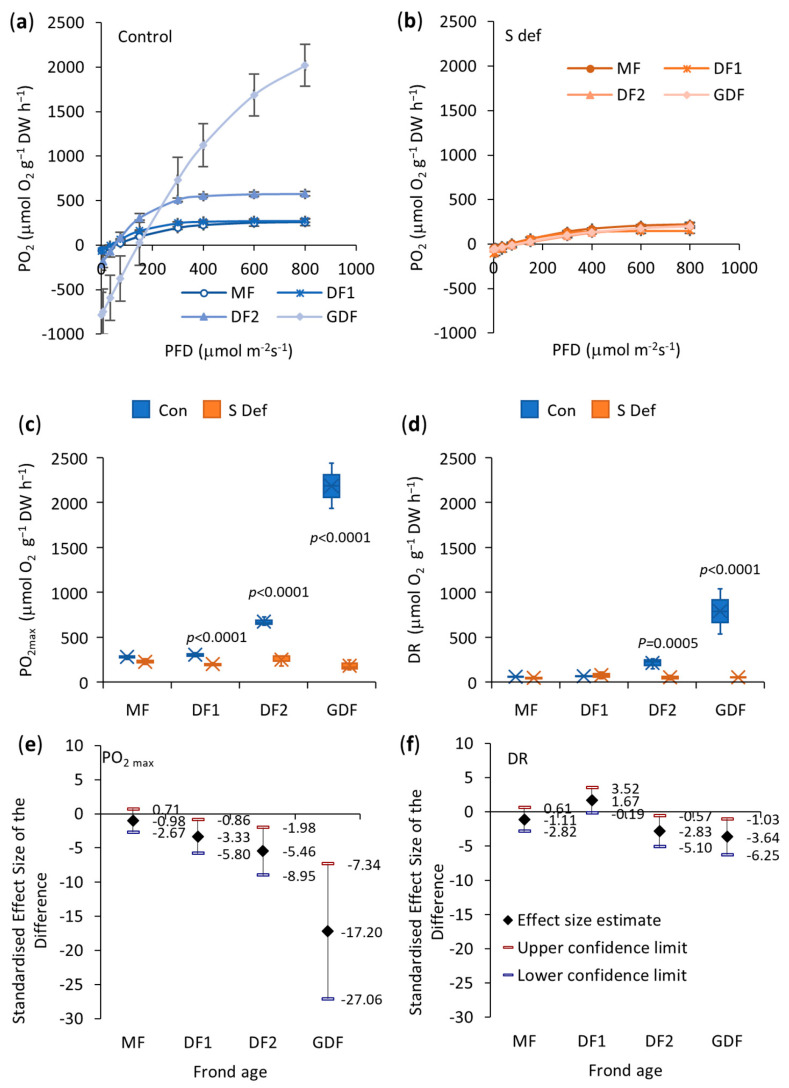
Net O_2_ production (μmol O_2_ g^−1^ DW h^−1^) in *S. polyrhiza* control fronds (**a**) and fronds subjected to S deficiency (**b**). Box plots of (**c**) maximum rate of net O_2_ production (PO_2 max_, μmol O_2_ g^−1^ DW h^−1^) and (**d**) dark respiration rate (DR, μmol O_2_ g^−1^ DW h^−1^) in both control and S-deficit conditions of *S. polyrhiza*. Estimates of the size of the difference (Hedges *d*) between treatment and control groups for PO_2 max_ (**e**) and DR (**f**). Frond age categories include mother fronds (MF, the oldest), first daughter fronds (DF1), second daughter fronds (DF2), and granddaughter fronds (GDF, the youngest). *p* values were obtained from the Kruskal-Wallis and post hoc Conover-Iman tests with Bonferroni correction (α = 0.0018) for differences between the control and S-deficit for each frond pair.

In control samples of MF, DF1, and DF2, the rate of net O_2_ production begins to slow down at a lower light intensity (~150 μmol m^−2^ s^−1^), reaching a saturation point at an irradiance 400 μmol m^−2^ s^−1^, a relatively low light level that avoids photodamage. In contrast, for the youngest fronds (GDF), the rate of net O_2_ production slows down above 400 μmol m^−2^ s^−1^, reaching a saturation point above 700 μmol m^−2^ s^−1^, suggesting that they can utilize more light energy. The light saturation point in S-deficient plants was reached at an irradiance level of 600 μmol m^−2^ s^−1^ in MF and DF2, at approx. 300 μmol m^−2^ s^−1^ in DF1, while the light saturation point in S-deficient GDF remained at the same light level (Figure 3a,b). However, the maximum O_2_ production rates did change (Kruskal-Wallis K = 20.71, df = 7, *p* = 0.004), Figure 3.

Post-hoc pairwise comparisons using the Conover-Iman test with the Bonferroni correction (α = 0.0018) showed significant differences between several group pairs. The oldest fronds, MF and DF1, had the lowest maximum net O_2_ production rate, with no significant difference observed between them (*p* = 0.374), suggesting functional similarity. Compared to their mother fronds, DF2 showed a significant 2-fold increase (*p* = 0.014), and GDF showed an 8-fold increase in maximal net O_2_ production rate (*p* = 0.006), indicating their higher photosynthetic efficiency (Figure 3c).

Older fronds (MF and DF1) also had relatively low and similar dark respiration rates (DR, μmol O_2_ g^−1^ DW h^−1^), with no difference observed between them (*p* = 0.330). However, second-generation daughter fronds showed a higher metabolic demand. DF2 showed a 3-fold increase (*p* = 0.002), and GDF showed a 12-fold increase (*p* = 0.002) in DR compared to their mother fronds (Figure 3d).

S deficiency did not affect the maximum rate of net O_2_ production in the oldest fronds (*p* = 0.065). However, it significantly decreased O_2_ production in younger fronds, with the most considerable reduction in the youngest GDF—an 11-fold decrease. In contrast, DF1 and DF2 each showed a 2-fold decline in the maximum rate of net O_2_ production under the S-deficit (Figure 3c,e). Furthermore, S deficiency did not significantly affect the dark respiration rate in mother fronds (MF and DF1), although DF1 showed a slight increase under S-deficit. However, younger fronds had a significantly reduced dark respiration rate under S-deficit—4-fold in DF2 and 14-fold in GDF (Figure 3d,f). Low respiratory rates require little photosynthesis to bring the net gas exchange rates to zero, allowing plants to survive in light-limited environments.

The light compensation points (LCP) for MF, DF1, and DF2 were at an irradiance of 46, 50, and 58 μmol m^−2^ s^−1^ in control and at 46, 52, and 62 μmol m^−2^ s^−1^ in S-deficient conditions, respectively. In contrast, the youngest fronds (GDF) had significantly higher LCP values than all other fronds in both control conditions (182 μmol m^−2^ s^−1^) and S-deficit conditions (108 μmol m^−2^ s^−1^). Therefore, only the LCP of GDF was significantly affected by S deficiency (*p* < 0.0001).

### 2.4. Rapid Light Curve

**Figure 4:**
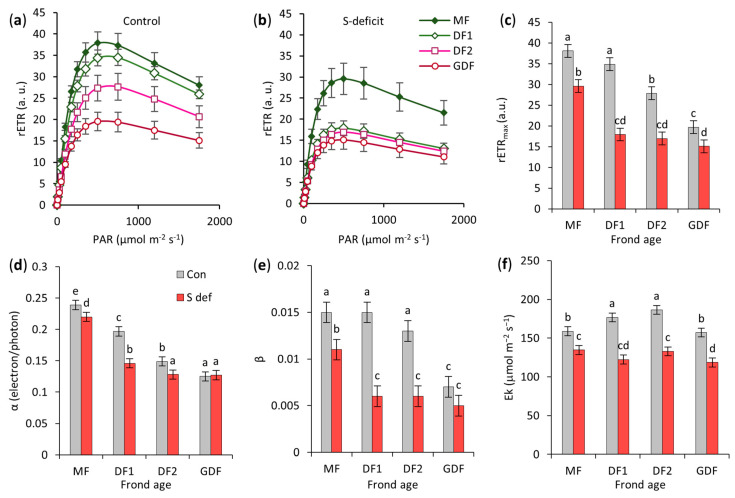
Effect of actinic irradiance on rapid light curves (RLC) with mean rETR (arbitrary units, dimensionless) of each category plotted with the standard deviation (n = 10) of *S. polyrhiza* fronds in control (**a**) and S-deficit conditions (**b**). Calculated mean values and 95% confidence interval (n = 10) of maximum electron transport rate (**c**) through PSII (rETR_max_), PSII photosynthetic efficiency at low light intensities –α (electron/photon, dimensionless) (**d**), PSII down-regulation capabilities at high light intensities—β (electron/photon, dimensionless) (**e**), and minimum saturating irradiance—Ek (μmol photons m^−2^ s^−1^) (**f**) under control and S-deficit conditions. Frond age categories include mother fronds (MF, the oldest), first daughter fronds (DF1), second daughter fronds (DF2), and granddaughter fronds (GDF, the youngest). Different letters indicate statistically significant differences between categories (two-way ANOVA and Tukey HSD, *p* < 0.05).

The electron sink and source capacities of the photosynthetic apparatus were evaluated from the F_0-M_ and F data associated with the Rapid Light Curve (RLC). The RLC results of *S. polyrhiza* grown under control and S-deficit conditions (Figure 4) showed distinctive patterns in almost all derived parameters.

Sulfur deficiency and frond age had a strong influence on rETRmax, as indicated by their large effect sizes (Table S2). S-deficit alone explained 36% of the total variance, and FA accounted for nearly half. In addition, a significant interaction was indicated, although it accounted for a smaller proportion of variance (7%), suggesting that the effect of the S-deficit depended to some extent on the FA (Table S2). In control conditions, the rETRmax through PSII was highest in older maternal fronds (MF and DF1), reflecting their fully developed photosynthetic apparatus, not significantly differentiated between them. The youngest fronds had the lowest rETRmax, which was twice as low as the value observed in MFs. The highest decrease in rETRmax was observed in DF1 (49%), while the lowest was in MF (22%) and GDF (23%), Figure 4c.

As for PSII’s photosynthetic efficiency at low light intensities—α, frond age was the dominant source of variation (ω^2^ = 0.822) with a very large effect, as opposed to S-deficit (ω^2^ = 0.065) and their interaction (ω^2^ = 0.042), which contributed to the total variance a lot less (Table S2). PSII’s photosynthetic efficiency was the highest in maternal fronds, indicating a fully developed photosynthetic apparatus, and the lowest in the youngest frond (GDF). Again, the largest impact of the S-deficit was on α of DF1, with a standardized effect size of 9.7 based on Tukey HSD post-hoc analysis of differences, representing a 26% decrease. At the same time, there was no effect on α of GDF, as the standardized effect size of the difference was 0.3 (*p* = 1), Figure 4d.

Sulfur deficiency, FA, and their interaction significantly affected PSII down-regulation capabilities at high light intensities—β (Table S2). The S-deficit accounted for the largest share of the variance (41%). FA also significantly influenced the variability of β (33%), while their interaction, although significant, contributed a smaller but meaningful fraction (10%). β decreased significantly in all fronds under S deficiency (Figure 4e), and in all daughter fronds (DF1, DF2, GDF), β dropped to the same level, indicating the activation of photoprotective mechanisms. However, the magnitude of that drop depended on the specific age, with the largest effect observed in DF1 (a 60% decrease; the standardized difference, or effect size, based on Tukey HSD analysis of differences, was 11.9).

Similar to previous parameters derived from RLC, S-deficit, FA, and their interaction significantly affected the minimum saturating irradiance—Ek (Table S2). S-deficit was the most influential variable, accounting for the largest share of the variance and effect size. The FA alone and its interaction with S-deficit accounted for a smaller proportion of the total variance with smaller effect sizes that were significant but showed no regularity or trend. This decrease ranged from 15% in MF to 31% in DF1. Therefore, in all S-deficient fronds, the transition from productive photosynthesis to protection via non-photochemical quenching occurred at an irradiance of approximately 120 μmol m^−2^ s^−1^. In contrast, in control conditions, this transition occurred at irradiance levels above 150 to 180 μmol m^−2^ s^−1^ for *S. polyrhiza* grown under our conditions (the level of usable light), as shown in Figure 4f.

Based on the rapid light curve, the older maternal fronds (MF and DF1) have a higher baseline photosynthetic capacity. However, under sulfur deficiency, DF1 experiences the deepest decline. Although the youngest fronds (GDF) had lower photosynthetic capacity, they were much less affected by the S-deficit, probably due to enhanced photoprotective mechanisms.

### 2.5. Redox State and Dynamics of Electron Transport Through PSI

**Figure 5:**
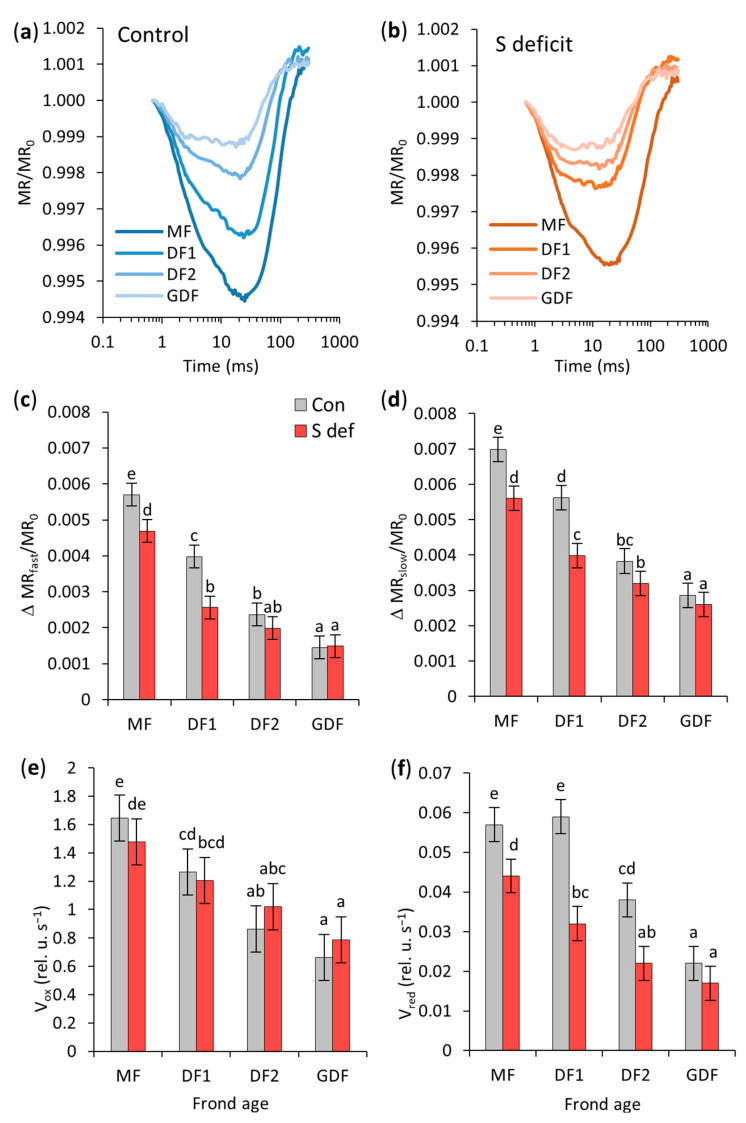
Modulated reflection at 820 nm (MR_820_ signal) normalized to MR_0_ in control fronds of *S. polyrhiza* (**a**) and fronds exposed to S-deficit (**b**). The time point 0.7 ms represents the first reliable value of the MR/MR_0_. Relative amplitudes of the fast phase, ΔMR_fast_/MR_0_ = (MR_0_ − MR_min_)/MR_0_ (**c**), and the slow phase, ΔMR_slow_/MR_0_ = (MR_max_ − MR_min_)/MR_0_ (**d**). The oxidation rate of plastocyanin (PC) and PSI reaction centers (P700)—V_ox_ (**e**) presented as the slope of the fast phase (from 0.7 to 2 ms), and PC^+^ and P700^+^ re-reduction rate—V_red_ (**f**) as the slope of the slow phase (from 40 to 100 ms). The values were presented as means with 95% CI (n = 10). Different letters indicate statistically significant differences between categories (two-way ANOVA and Tukey HSD, *p* < 0.05).

The data in Figure 5 show MR_820_ signals normalized by MR_0_ (0.7 ms) and were used to analyze the redox state and electron transport dynamics of photosystem I (PSI) reaction center (P700) and PC in *S. polyrhiza* fronds of different ages (MF, DF1, DF2, GDF) in control conditions and under S-deficit.

ΔMR_fast_ and ΔMR_slow_ provide insight into whether PSI is effectively re-reduced after oxidation or remains oxidized due to slower electron donor activity. The amplitudes of the fast (ΔMR_fast_/MR_0_) and slow phases (ΔMR_slow_/MR_0_) increased significantly with age in both control and S-deficit conditions (Table S3, Figure 5c,d). More than 80% of the variability in the fast phase data was attributed to FA, with a very strong effect, while S-deficit and the FA × S interaction contributed less (Table S3).

Frond age was also the most influential variable in the slow phase (Table S3). Like in the fast phase, S-deficit and interaction were less represented in the total variance. Mother fronds had 3–4 times higher ΔMR_fast_ and ~2.4 times higher ΔMR_slow_ than the youngest GDF in control and S-deficit conditions, respectively. Under the S-deficit, ΔMRfast significantly decreased in MF (18%) and DF1 (36%) but remained unchanged in DF2 and GDF. Similarly, ΔMR_slow_ declined by 20% in MF and 29% in DF1 under S-deficit and did not differentiate in DF2 and GDF (Figure 5c,d).

Statistical analysis showed that the regression model predicting V_ox_ using S-deficit, FA, and their interaction, although significant overall (*p* < 0.0001), remained unaffected by S-deficit (*p* = 0.7), Table S3. Frond age was the only significant variable with a large effect size, accounting for a significant portion of the explained variance. Therefore, the rate of initial photooxidation of P700 (V_ox_) increased with age, with MF exhibiting 2.5- and 2-fold higher V_ox_ than GDF under both control and S-deficit conditions (Figure 5e).

The re-reduction rate (V_red_) of PC^+^ and P700^+^ was also higher in maternal fronds (2.6 times higher than in their daughter fronds under both control and S-deficit conditions). Frond age was also the most influential variable (57% of total variation) with a large effect size, but S-deficit also had a significant effect (Table S3). Although their interaction was significant, its contribution was lower (5%) with a smaller effect size. Consequently, the S-deficit decreased V_red_ by 23% in MF, 46% in DF1, and 42% in DF2, while the re-reduction rate of P700^+^ in GDF remained unaffected (Figure 5f).

The total amount of photo-oxidizable P700 was estimated from ΔMR_max_, and frond age was the most influential variable (Table S3). In control conditions, the ΔMR_max_ progressively decreased from older to younger fronds. ΔMR_max_ values were 79.4 (75.1–83.7) in MF, 67 (62.7–71.3) in DF1, 55.2 (50.9–59.5) in DF2, and 34.7 (30.4–39.0) in GDF. S-deficit significantly decreased ΔMR_max_ in MF by 17%, in DF1 by 29%, and in DF2 by 32%. However, the S-deficit did not affect ΔMR_max_ in GDF, as the average value remained at 35.

Overall, frond age was the dominant factor affecting PSI function, with older maternal fronds having significantly higher amplitudes in ΔMR_fast_ and ΔMR_slow_ than younger fronds in both control and S-deficit conditions. Sulfur deficiency significantly impacted PSI function in older fronds, especially DF1.

### 2.6. Prompt Chlorophyll Fluorescence Induction Curves

**Figure 6:**
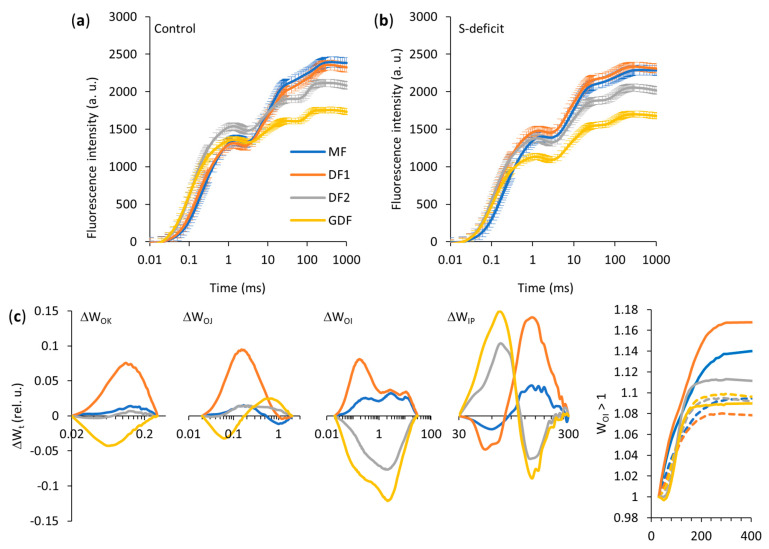
Prompt chlorophyll fluorescence curves (OJIP) of *S. polyrhiza* fronds in control (**a**) and S-deficit (**b**) conditions. Difference kinetics of relative variable fluorescence induction curves normalized by F_0_ and F_K_ (- O–K phase), F_0_ and F_J_ (O–J phase), F_0_ and F_I_ (O–I phase), and F_I_ and F_P(M)_ (I–P phase), and calculated as ΔW_t_ = W_t(S def)_ − W_t(control)_ and W_OI_ ≥ 1 curve in the linear time range (full lines represent fronds in control conditions, and dashed lines in S-deficit) (**c**). Frond age categories include mother fronds (MF, the oldest), first daughter fronds (DF1), second daughter fronds (DF2), and granddaughter fronds (GDF, the youngest). Each curve is the mean of 10 measurements (±SEM in the OJIP curve).

Under control conditions, two mother fronds (MF and DF1) showed similar OJIP curves. However, the younger daughter fronds (DF2 and GDF) differed from their mother’s curves (from F_0_ to F_P(M)_) and each other (from K- to P-step). S deficiency altered the fluorescence transients of each frond differently (Figure 6a,b). To highlight these differences, fluorescence curves were double normalized at various time scales, as shown in Figure 6c.

The ΔW_OK_ difference kinetics revealed a positive L-band in DF1 under S-deficit, suggesting lower energetic connectivity, whereas GDF showed a negative L-band, indicating higher cooperativity of PSII units. The ΔW_OJ_ difference kinetics also revealed only a positive K-band in DF1, which could be related to the imbalance in electrons leaving and arriving at the PSII reaction center. GDF showed biphasic kinetics with faster early electron donation, which later shifted to the acceptor-side bottleneck, likely leading to QA^−^ accumulation or slower transport from the donor side.

Further downstream in the electron flow, the ΔW_OI_ difference kinetics revealed a significant increase in the J-band due to S deficiency in maternal fronds (MF and DF1) and a decrease in fluorescence in the J-band of younger daughter fronds (DF2 and GDF). The W_OI_ ≥ 1 curve in the linear time range showed differences in the I-P phase among these fronds, providing information about the pool size of the PSI-end electron acceptors (Figure 6c). The most significant decrease in amplitude was observed in DF1. In contrast, GDF showed a minor increase in W_OI_ ≥ 1 curve amplitude. Accordingly, the S-deficit reduced the pool size of end electron acceptors on the PSI acceptor side in older fronds, especially DF1. To determine any change in the overall rate constant for reducing the pool of end electron acceptors (independent of possible effects on its size), difference kinetics normalized between the I- and P-steps, as ΔW_IP_, were analyzed. The time it takes half of the PSI-end electron acceptors to be reduced was estimated at the half-time of the curve rise (W_IP_ = 0.5). Under S-deficit conditions, only DF1 showed a 12% reduction in this half-time (from 109 to 96 ms), suggesting faster electron transfer and a higher PSI-end electron acceptor reduction rate. This half-time did not change under S-deficit conditions in all other fronds.

However, all fronds exhibited biphasic ΔW_IP_ difference kinetics, indicating dynamic but different adjustment strategies in the terminal stages of photosynthetic electron transport. In older fronds (MF, DF1), the positive peak began slightly later (~80–300 ms), indicating that the S-deficit strongly alters the IP phase, especially in DF1, disrupting electron flow beyond the PSI acceptors. In younger fronds (DF2, GDF), an inflection point near 110 ms, from positive to negative amplitudes, indicates a smaller or delayed IP phase under the S-deficit.

### 2.7. Effect of S-Deficit and Frond Age on JIP Test Parameters

**Figure 7:**
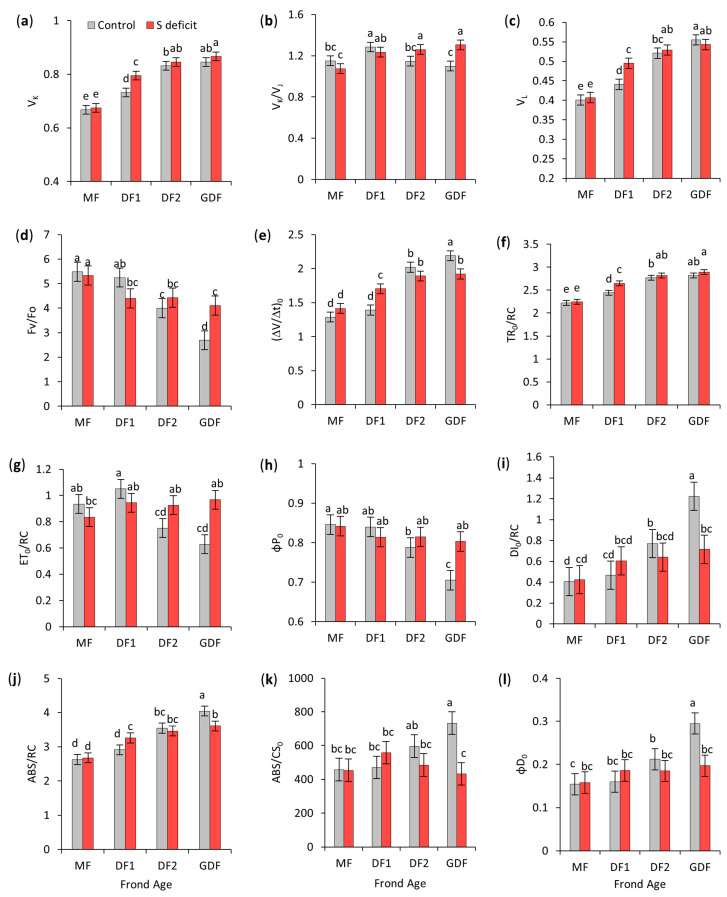
Mean values with 95% CI (n = 10) of JIP test parameters characteristic for PSII donor side (**a**,**b**), the performance of PSII reaction centers (**c**–**i**) and antenna complex properties (**j**–**l**) in *S. polyrhiza* four frond colony grown in control and S-deficient conditions. Frond age categories include mother fronds (MF, the oldest), first daughter fronds (DF1), second daughter fronds (DF2), and granddaughter fronds (GDF, the youngest). Different letters indicate statistically significant differences between categories (two-way ANOVA and Tukey HSD, *p* < 0.05).

**Figure 8:**
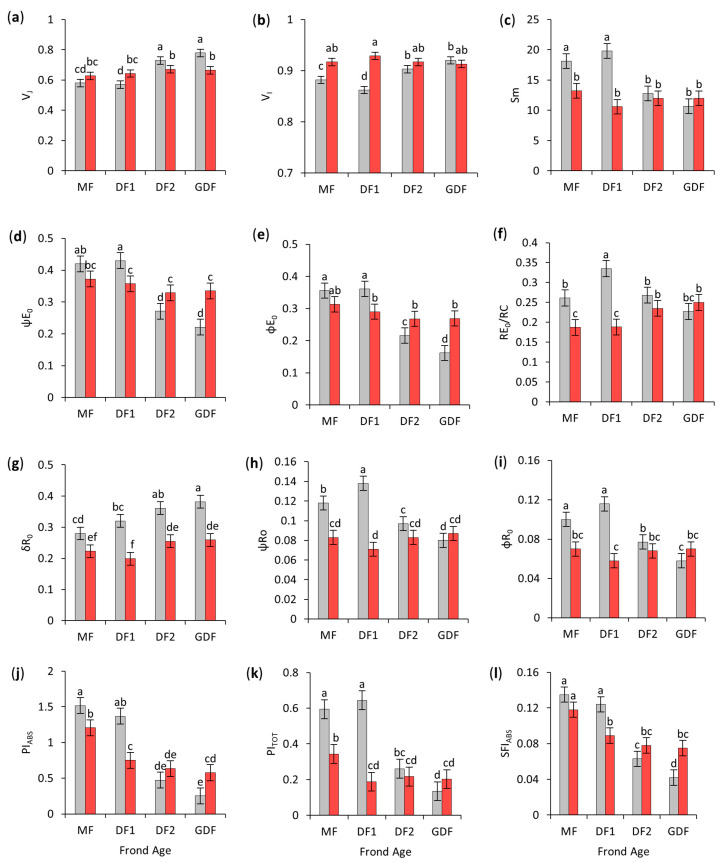
Mean values with 95% CI (n = 10) of JIP test parameters characteristic for PSII acceptor side (**a**–**e**), PSI–related parameters (**f**–**i**), and plant performance indices (**j**–**l**) in *S. polyrhiza* four frond colony grown in control and S-deficient conditions. Frond age categories include mother fronds (MF, the oldest), first daughter fronds (DF1), second daughter fronds (DF2), and granddaughter fronds (GDF, the youngest). Different letters indicate statistically significant differences between categories (two-way ANOVA and Tukey HSD, *p* < 0.05).

**Figure 9:**
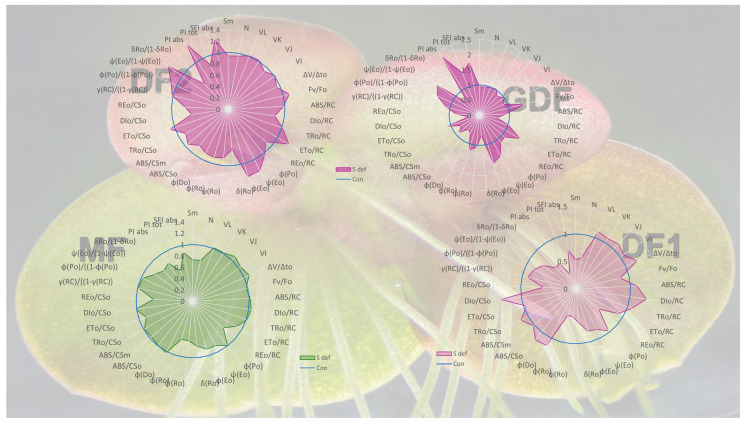
Radar plots of selected JIP test parameters of chlorophyll *a* fluorescence transient in *S. polyrhiza* fronds under S-deficit conditions normalized to the control. Frond age categories include mother fronds (MF, the oldest), first daughter fronds (DF1), second daughter fronds (DF2), and granddaughter fronds (GDF, the youngest).

JIP test parameters were further analyzed to evaluate photosynthetic performance, focusing on potential alterations in electron transport efficiency between PSII and PSI in *S. polyrhiza* fronds of different ages under S-deficient conditions (Figure 7, Figure 8 and Figure 9). According to two-way ANOVA, frond age was the primary driver of changes in chlorophyll fluorescence parameters (e.g., F_v_/F_0_, V_L_, V_K_, V_J_, ABS/RC, DI_0_/RC, TR_0_/RC, φP_0_, ψE_0_, φE_0_, δR_0_, φD_0_, PI_ABS_, PI_TOT_, SFI_ABS_), indicating that developmental changes strongly influence primary reactions of photosynthesis (Table S4).

Although the S-deficit was significant for many parameters (e.g., V_L_, V_K_/V_J_, S_m_, ABS/CS_0_, DI_0_/RC, ET_0_/RC, φP_0_, φD_0_, PI_ABS_, PI_TOT_), its effect size was relatively smaller than that of the frond age. Furthermore, for some parameters (e.g., F_v_/F_0_, V_J_, ψE_0_, φE_0_, and SFI_ABS_), S-deficit alone was not significant. However, its interaction with frond age was significant with a large effect size, indicating that the impact of S-deficit on those parameters depends on the fronds’ age, not uniformly across all fronds but instead influencing specific fronds.

A pairwise post-hoc Tukey HSD test was performed to compare frond ages and treatment levels, and results for relevant parameters are presented in Figure 7 and Figure 8.

#### 2.7.1. PSII Donor Side

Potential disturbances or inefficiencies on the donor side of PSII were evaluated within the K-step: V_K_ progressively increased from the oldest to the youngest fronds, with GDF exhibiting 27–28% higher VK than MF in both control and S-deficit conditions. S deficiency significantly increased V_K_ only in DF1 (for 9%). The ratio V_K_/V_J_ was very stable across all fronds in control conditions, ranging from 1.1 in DF2 and GDF to 1.3 in DF1, indicating similar donor-side efficiency. However, under S-deficit conditions, V_K_/V_J_ increased by 10% in DF2 and by 20% in GDF, indicating either a higher K amplitude or a suppressed J step, possibly indicating instability on the PSII donor side of younger fronds or changes in functional antenna size (Figure 7a,b).

#### 2.7.2. Antenna Complex Properties

ABS/RC evaluated the size of the light-harvesting antennas relative to reaction centers. This functional antenna size increased from mother fronds towards the youngest fronds progressively in both control and S-deficit conditions (e.g., GDF had a 54% higher ABS/RC than MF in control and 35% higher than MF in S-deficit conditions), indicating larger functional antennas in younger fronds, only in GDF (by 41%), suggesting that the youngest fronds adjust their overall antenna size under S deficiency (Figure 7k).

The S-deficit increased ABS/RC in DF1 (12%) but decreased it by 11% in GDF, highlighting different responses between DF1 and GDF (Figure 7j). Similarly, ABS/CS_0_ increased in control conditions from maternal fronds to the youngest fronds, with GDF having 60% higher ABS/CS_0_ than MF, and S-deficit decreased ABS/CS_0_.

Tracking DI_0_/RC over time as fronds mature can reflect adjustments in antenna size as fronds balance energy capture with the need to prevent photodamage. In control conditions, DI_0_/RC was the highest in the youngest GDF and the lowest in the oldest MF, with GDF having 202% and DF2 90% higher DI_0_/RC than their maternal fronds, DF1 and MF, respectively. This increase in younger fronds was a sign that absorbed light energy exceeded the capacity for its utilization in photochemistry, so to prevent overexcitation of PSII and potential photodamage, due to lower electron transport capacity, energy was dissipated. Under S deficiency, DI_0_/RC significantly decreased (by 42%) in GDF, indicating a change in the processing of absorbed light by reorganizing light-harvesting capacity (Figure 7i).

The maximum quantum yield of nonphotochemical deexcitation (φD_0_), which quantifies the energy dissipated as heat by the antenna system, therefore, increased from older to younger fronds and was significantly higher in DF2 (by 38%) and in GDF (by 84%) than in their maternal fronds. Under S-deficit, φD_0_ decreased only in the youngest frond (GDF) by 33% (Figure 7l).

#### 2.7.3. Performance of PSII Reaction Centers

Variable fluorescence at the L-band (V_L_)_,_ linked to the connectivity of PSII units, also progressively increased from the oldest to the youngest fronds, with GDF having 33–38% increased V_L_ than older fronds in both control and S-deficit conditions. On the other hand, S deficiency significantly increased V_L_ only in DF1 (by 12%), Figure 7c.

The maximum efficiency of the oxygen-evolving complex and excitation energy in PSII (F_V_/F_0_) in control conditions also progressively decreased from MF to GDF, with GDF having 57% lower F_V_/F_0_. Under the S-deficit, F_V_/F_0_ decreased by 16% in DF1, showing impairment, and increased by 53% in GDF, suggesting improved use of excitation energy (Figure 7d).

ΔV/Δ(t)_0_ or M_0_, the initial slope of relative variable fluorescence that indicates the net closing rate of the reaction centers, was 57% higher in younger DF2 and GDF than in their maternal fronds. S deficiency led to a significant increase of M_0_ in DF1 (23%) and a significant decrease in the youngest GDF (12%), Figure 7e.

Furthermore, the amount of energy trapped by each reaction center (TR_0_/RC) increased from oldest to youngest fronds in both control and S-deficit conditions (up to 25% in GDF), making frond age the most influential variable and confirming a larger and more effective antenna size capable of capturing more energy in younger fronds. Simultaneously, ET_0_/RC decreased from the oldest to the youngest fronds in control conditions (e.g., GDF had 33 and 40% lower ET_0_/RC than MF and DF1), Figure 7g. Under the S-deficit, TR_0_/RC increased significantly only in DF1 (8%), while ET_0_/RC significantly increased in DF2 and GDF by 23 to 54%, respectively (Figure 7f,g).

The overall efficiency of absorbed light energy used in PSII photochemistry decreased significantly from the older to younger fronds, with GDF exhibiting a 17% lower φP_0_ than MF. However, under the S-deficit, φP_0_ increased by 14% only in GDF (Figure 7i).

#### 2.7.4. PSII Acceptor Side Parameters

The increase in V_L_ was consistent with an increase in V_J_, which was also significantly higher in younger daughter fronds (DF2 and GDF) than in older fronds, by 28 and 34%, respectively, reflecting higher QA^−^ accumulation in younger fronds. Under S deficiency, V_J_ significantly increased in DF1 (by 13%), indicating that electron flow to the acceptor side exceeded the electron flow from the donor side. On the contrary, V_J_ decreased by up to 15% in DF2 and GDF, suggesting a decrease in electron flow from the donor side, which restricted QA accumulation in its reduced form, resulting in lower net closure of reaction centers (Figure 8a).

Similarly, the efficiency (ψE_0_) and quantum yield (φE_0_) of electron transport from QA to the PQ pool were reduced by 35–49% and 39–55%, respectively, in the younger fronds (DF2 and GDF) (Figure 8d,e). Under the S-deficit, ψE_0_ increased significantly in DF2 (21%) and GDF (52%). At the same time, φE_0_ decreased by 20% in DF1 but increased by 24% in DF2 and 66% in GDF, relative to their respective controls.

The number of non-reducing PSII reaction centers (V_I_) in control conditions was significantly higher in younger daughter fronds (up to 7%) than in maternal fronds, probably because of immature PSII or higher turnover rates. Under the S-deficit, V_I_ significantly increased only in maternal fronds (4% in MF and 8% in DF1), indicating the accumulation of non-functional RCs (Figure 8b). However, the proxy for the number of electron carriers per electron transport chain, S_m_, was significantly higher (30–46%) in maternal fronds (MF and DF1) than in their daughter fronds (DF2 and GDF). S deficiency caused a significant decrease in S_m_ only in maternal fronds (27% in MF and 47% in DF1), indicating a greater impact on the electron carrier capacity of older tissues (Figure 8c).

Overall, these results indicated that younger fronds activate adaptive mechanisms to enhance electron transport under S-deficit, while DF1 experiences an imbalance of electron flow on the acceptor side of PSII.

#### 2.7.5. PSI-Related Parameters

The rate at which electrons are transported from reduced carriers to the end electron acceptors per reaction center RE_0_/RC indicated that DF1 had the most efficient electron transport beyond PSII in control conditions. S deficiency decreased RE_0_/RC in MF by 28% and DF1 by 44%, while in younger fronds, RE_0_/RC was unaffected by S-deficient conditions (Figure 8f).

In contrast, the probability of electron transport from reduced intersystem carriers (PQH_2_) to the terminal PSI acceptors (δR_0_) increased significantly from the oldest to the youngest fronds, with GDF having 36% higher δR_0_ than MF. However, the efficiency of electron transport from reduced QA (ψR_0_) and the net yield of electron transfer from PSII to final PSI acceptors (φR_0_) were significantly lower by 18% and 42% for ψR_0_ and 23% and 50% for φR_0_ in DF2 and GDF relative to their maternal fronds. S deficiency significantly decreased δR_0_ across all fronds, ranging from a 20% decrease in MF to 38% in DF1. As for the efficiency (ψR_0_) and quantum yield (φR_0_) of electron transport to end electron acceptors, they declined by 30% and 50% in MF and DF1 under S deficiency but remained unaffected in younger fronds (Figure 8g–i).

#### 2.7.6. Overall Plant Performance

Considering all the above factors, frond age was the most influential variable affecting SFI_ABS_, PI_ABS_, and PI_TOT_, explaining most of the observed variance among fronds. Younger fronds (DF2 and GDF) showed significantly lower values of SFI_ABS_ (53–66%), PI_ABS_ (69–82%), and PI_TOT_ (56–79%) than MF and DF1, highlighting the developmental influence on both structural and performance indices.

Under S-deficit conditions, SFI_ABS_ decreased by 28% in DF1 but increased by 79% in GDF; PI_ABS_ significantly decreased in MF (by 21%) and DF1 (by 45%) but increased by 128% in GDF; and PI_TOT,_ likewise, decreased in MF (42%) and DF1 (71%) and increased by 50% in GDF, but due to the high confidence interval of the difference, this increase was not statistically significant (*p* = 0.607), Figure 8j–l.

The JIP test results summarized in Figure 9 demonstrated that S deficiency negatively impacts older fronds, especially DF1, with declines in electron transfer efficiency and performance indices. Younger fronds (DF2 and especially GDF) show adaptive traits like increased electron transfer efficiency and decreased energy dissipation, retaining or even improving specific photochemical parameters under S-deficit conditions.

## 3. Discussion

### 3.1. Age-Dependent Variation in Chloroplast Ultrastructure of S. polyrhiza

The *S. polyrhiza* four-frond colony consists of three generations of varying ages. Mother fronds (MF) produce daughter fronds (DF1, DF2) by vegetative budding from one or two meristematic pockets. These daughter fronds then bud off to form the next generation of daughters, known as granddaughters, or GDFs [21,27,28]. Mother fronds are the metabolic center of the colony, with numerous large plastoglobuli, which are characteristic of high metabolic activity and/or the onset of the senescence process. As a transient storage of energy-rich lipids, these supersized plastoglobuli can serve as carbon and energy sources for developing daughter fronds [29], facilitating the synthesis and recycling of, for example, phylloquinone, plastoquinone-9, plastochromanol-8, and tocopherol, as well as their exchange with the thylakoid membrane for redox regulation of the photosynthetic process [30,31,32,33]. Because extensive thylakoid networks in MFs with compact grana stacks maximize electron transport efficiency, they favor the maintenance of the structure and the redistribution of nutrients, with a reduced focus on maximizing their photosynthetic output.

Compared with its mother frond, the first-generation daughter fronds (DF1) have numerous but smaller plastoglobuli, indicating higher current metabolic activity but less dependence on extensive lipid remodeling. At the same time, well-defined thylakoids reflect active photosynthesis correlating with efficient light harvesting, while a smaller amount of accumulated starch suggest its transitional phase where photosynthesis supports its own growth and contributes to the colony’s resources by creating a new frond. Only two-to-three thylakoid layers in the youngest fronds (GDF) maximize light absorption and support high photosynthetic requirements during the rapid growth. Large starch granules in GDF and DF2 reflect significant energy storage required for intense metabolism. At the same time, stromules on chloroplasts and tubular protrusions on mitochondria suggest active inter-organelle communication [34,35]. Numerous small lipoprotein plastoglobuli associated with thylakoids enable dynamic lipid exchange [29]. Overall, these features are typical for growth-oriented fronds. Therefore, although minimal, these developmental differences could modulate each frond’s response to sulfur deficiency.

### 3.2. Pigments, Total Anthocyanins, and Starch

Each frond’s developmental and functional role within the colony is reflected in its concentrations of starch and chlorophyll under optimal conditions. While mature fronds (MF and DF1) have less chlorophyll, emerging fronds (DF2 and GDF) require higher chlorophyll content to enhance energy absorption. Under S deficiency, chlorophyll content decreased in all fronds, with the largest decrease in the younger ones. Many enzymes involved in pigment biosynthesis require sulfur-containing amino acids or depend on Fe-S clusters, and sulfur deficiency in young fronds reduces the activity of these enzymes and, consequently, pigment synthesis [36,37]. Furthermore, a glutathione pool is required for chlorophyll biosynthesis, which is decreased by S deficiency, especially in young plants [38]. Additionally, increased oxidative stress may damage photosynthetic pigments, leading to their degradation [39,40,41], a process that could have occurred under S deficiency.

The fixed carbon allocated during normal growth conditions (light, all nutrients present) is typically 25–30% toward transient starch and 7% toward soluble sucrose, and the rest is consumed in metabolic processes for growth and respiration [42]. To maximize plant growth and avoid acute carbon starvation, the transient starch synthesis and breakdown rate must match plants’ photosynthetic capacity in their current environment [43,44,45]. Mother fronds generally maintain a lower starch content because they prioritize supporting the colony’s energy needs over accumulating reserves. Meanwhile, younger fronds accumulate more starch, which correlates with their higher pigment content, increased photosynthetic activity, and more intensive metabolic requirements.

Under S deficiency, in older maternal fronds, transient starch increased two-fold, and in the youngest granddaughter fronds, five-fold. Why did starch accumulate considerably in sulfur-deficient conditions, especially in the youngest *S. polyrhiza* fronds? The phosphate/triose-phosphate (PTP) translocator maintains an optimal phosphate pool for ATP production by exchanging triose-phosphates produced in the Calvin Benson Basham cycle for inorganic phosphates (Pi) from the cytosol [46]. In the youngest frond, the respiration rate decreased to a level similar to that of older maternal fronds, meaning less carbon was metabolized for energy, diverting more carbon to starch synthesis under S-deficit. S deficiency slows down major cytosolic metabolic pathways [7,10,47], lowering triose-phosphate consumption, decreasing Pi availability, and compromising the function of the PTP translocators. As a result, chloroplast stroma faces phosphate depletion, threatening photosynthetic efficiency and ATP production. By redirecting triose-phosphates toward starch synthesis, Pi is recycled back into the stroma [42,48], sustaining ATP synthase activity and allowing photosynthesis to continue despite low sulfur. According to Sun et al. [6], sulfur deficiency in *S. polyrhiza* enhances the expression of key enzymes involved in starch synthesis, including ADP-glucose pyrophosphorylase (AGPase) and granule-bound starch synthase (GBSS). Wang et al. [49] determined that overexpression of AtPSP1, which encodes phosphoserine phosphatase, enhances starch accumulation in *Lemna turionifera* 5511 under sulfur-limiting conditions. Hence, in S-deficient conditions, starch accumulation was likely a result of the upregulation of genes associated with starch synthesis and the downregulation of genes associated with starch degradation. A similar response was determined for *Chlorella sorokiniana* under S deficiency [50]. Therefore, combined with reduced respiration, starch synthesis and degradation enzymes upregulation and downregulation resulted in significant starch accumulation [6,10,51].

Colonies grown in the control media also showed a slight gradation of anthocyanins from maternal to younger fronds. Younger fronds are still growing, and higher levels of anthocyanins are likely needed to protect themselves from potential photodamage or mitigate oxidative stress due to growth prioritization [52,53]. Therefore, anthocyanin accumulation under optimal conditions appears age-dependent, likely increasing in younger fronds to help them cope with stress.

Plants experiencing sulfur deficiency undergo various physiological and biochemical changes, and one of the most noticeable responses is a decline in growth rate. In this experiment, the growth of *S. polyrhiza* declined by 33% (per surface area, not by frond number) after 6 days of exposure to S-deficit (Figure S1). The main cause of this decrease was the sulfur-deficient plants’ inability to synthesize sufficient proteins and lipids necessary for all cellular functions while, at the same time, sustaining fast growth, one of the fastest growth rates in higher plants [13].

When the energy supply is limited, like under sulfur deficiency, light assimilation capability is reduced, and normal light can be perceived as high light. It is well known that high light induces the expression of phenylpropanoid and flavonoid biosynthetic enzymes, leading to the production of anthocyanins [54,55,56]. In this study, anthocyanin and starch levels in sulfur-deficient conditions skyrocket in the younger fronds. Anthocyanins can complement xanthophyll in the photoprotection of leaves during ontogenesis [57,58]. However, because anthocyanins in *S. polyrhiza* accumulate in the abaxial part of the frond, their photoprotective role may be attenuated from the typical adaxial accumulation pattern, and their exact role becomes unclear. On the one hand, anthocyanins can act as optical buffers of light reflection from the water’s surface [53,59] or, on the other hand, regulate light absorption under fluctuating light intensities [60]. Nevertheless, under high light, they accumulate on the adaxial surfaces to reduce photoinhibition of abaxial cells [60,61].

Although anthocyanin plasticity is an adaptive feature in high light or during light-sensitive developmental stages, its functional significance is an open debate [62]. It depends on plant species, the location of anthocyanin in the leaf or the leaf itself, and many other environmental conditions [57,63,64]. Perhaps anthocyanins in *S. polyrhiza* accumulate to mitigate photooxidation. Indeed, anthocyanins can scavenge superoxide radicals generated by chloroplasts, indicating that cytosolic anthocyanins can serve as effective antioxidants [65] and compensate for the lower antioxidant enzyme pool in young red leaves of *Photinia×fraseri*, making them more resistant to the production of superoxide radicals than mature green leaves [66]. However, none of these fit entirely into explaining anthocyanin hyperaccumulation on the abaxial surface of young *S. polyrhiza* plants under S-deficit.

On the other hand, anthocyanin accumulation could reflect carbon overflow due to growth limitation in an S-deficient environment. Excess sugar can trigger anthocyanin biosynthesis [67,68,69] since they act as carbon sinks to buffer sugar levels, preventing transitory sugar accumulation [62]. For example, the metabolic flux of *Landoltia punctata* under nutrient starvation was mainly directed into the starch and anthocyanin biosynthesis [70]. Li et al. [71] determined that sucrose blocks GA-mediated degradation of DELLA proteins, inhibiting plant growth while activating MYB75 gene expression and promoting anthocyanin biosynthesis, which might explain anthocyanin accumulation and growth arrest in stress conditions associated with high sucrose levels to either delay maturation, maintain firmness, or avoid premature abscission [72]. In *S. polyrhiza*, such a mechanism could explain why young fronds shift their metabolism into a non-growing, metabolite-storing state under S-deficient conditions. Preventing early frond abscission and extracting nutrients from their mother fronds ensures survival during extended nutrient scarcity or until the transition towards turions is complete. According to Acosta et al. [28] and Ziegler [21], mild or transient nutrient limitations may slow down duckweed growth and reduce daughter frond size, while pronounced starvation triggers turion development as the dominant survival strategy.

### 3.3. Photosynthesis

Older fronds had lower rates of photosynthesis and respiration, which were not affected by S deficiency since they had ceased growing and were in a phase of maintaining essential functions with reduced metabolic activity. Internal reserves of sulfur compounds accumulated earlier probably made mother fronds less sensitive to S deficiency, maintaining near-normal metabolic rates, indicating their stable but lower metabolic plasticity and reliance on stored reserves.

Under control conditions, the youngest fronds are actively growing and differentiating, which requires substantial energy for cell division, expansion, and the synthesis of cellular components. High photosynthesis and respiration rates meet this increased energy demand, providing ATP and a carbon skeleton. GDF’s high LCP reflects intense metabolism since a large proportion of carbon flows into the synthesis of amino acids [73]. However, sulfur deficiency lowers GDF’s metabolic requirements by slowing down the synthesis of sulfur compounds, which is reflected in a lower respiration rate and LCP, and is regulated by the demand for amino acids or sugars. This reduction adjusts the metabolic activity of GDF to preserve the energy needed for survival in the form of accumulated starch and anthocyanins. Therefore, the youngest fronds show greater metabolic plasticity, adapting to stress caused by S deficiency. Nevertheless, the maximum net photosynthetic rate in younger fronds was also highly reduced. A detailed analysis of photosynthetic performance, focusing on potential alterations in electron transport efficiency and balance between PSII and PSI, was assessed to explain this reduction by using three approaches: (1) Rapid Light Curves (RLC) derived from chlorophyll fluorescence parameters [74,75,76], (2) OJIP transients and related JIP test parameters [77,78], and (3) 820 nm absorbance/transmission measurements [79,80,81]. Together, these methods provided a comprehensive insight into the functioning of the photosynthetic apparatus in the *S. polyrhiza* four-frond colony under S-deficit.

### 3.4. Rapid Light Curve (RLC)

In control conditions, the older maternal fronds of *S. polyrhiza* (MF and DF1) had higher baseline photosynthetic capacity (rETRs), reflecting a more mature photosynthetic apparatus and supporting the observations of fully developed thylakoids and enzyme complements. Compared to older fronds, younger fronds (DF2 and especially GDF) had not yet reached this level of maturity and achieved their maximal photosynthetic potential. This is consistent with the findings of Bielczynski et al. [82], which determined that leaf and plant age is a key factor influencing the photosynthetic activity of Arabidopsis leaves during development, and with Bąba et al. [83], which showed that tor grass (*Brachypodium pinnatum*) expansion is related to photosynthetic performance during the light phase, with older strands being well acclimatized with lower photosynthetic performance, while younger strands display high-speed photosynthesis maximizing photosynthetic rates.

The results show that DF1 was the most vulnerable to S deficiency among all fronds, displaying the largest drops across all RLC-derived parameters despite its relatively high baseline capacity. Rattan [84,85] determined that nutrient deficiency affects physiological functions, which can be observed through decreased rETR and its correlation with irradiance. Although the youngest fronds (GDF) had a lower overall capacity (rETRmax), they maintained stronger photoprotection (no change in β) under higher irradiances. Thus, the S-deficit affected them much less, confirming earlier studies [82,86]. Moreover, all fronds shifted to photoprotection under S deficiency at lower irradiances, indicated by decreased Ek values. According to Henley [87], Ek is related to quenching, where photochemical quenching dominates below Ek, while nonphotochemical quenching dominates above its value. Harrison and Smith [88] similarly observed that nutrient deficiency decreases saturation irradiance rather than altering the initial slope (α) of the rETR–irradiance curve. Therefore, RLC demonstrated that the interaction between frond age and S availability led to distinct photosynthetic responses, with DF1 again emerging as the transitional frond most impacted by the S-deficit and GDF being more resilient.

### 3.5. Modulated Reflection—MR_820_

Modulated reflection at 820 nm (MR_820_) revealed kinetic constraints of the electron transport chain through PSI, how fast PSI can be driven to full oxidation P700^+^, and how quickly it can re-reduce [80,89,90,91]. Frond age was the dominant factor governing the PSI redox state and electron transport dynamics. The extent of P700 and PC oxidation and re-reduction increased with age, reflecting stronger PSI capacity, larger carrier pools (PC, P700, ferredoxin, etc.), and strong and stable electron flow in older maternal fronds. Although maternal fronds had a faster PSI oxidation rate (V_ox_), tied to a larger donor pool and more robust PSI than the younger fronds, V_ox_ remained unchanged across all fronds under the S-deficit, meaning that the initial photooxidation was not the bottleneck under sulfur deficiency. In some other stress conditions, V_ox_ did not decrease either. For example, Dąbrowski et al. [92] found no decrease in V_ox_ under drought stress in perennial ryegrass, and El-Mejjaouy et al. [93] observed a similar response in wheat plants under phosphorus deficiency conditions, while Schansker et al. [89] suggested that the fast phase significantly changes only when influenced by intense stress.

Following oxidation, PSI was effectively re-reduced in maternal fronds, indicating a more functional P700 and PC pool. Higher V_red_ in older fronds implies a stronger electron supply to PSI from cytochrome b_6_f, suggesting that re-reduction becomes faster as fronds age, which agrees with the findings of Gao et al. [90]. This is essential for sustaining linear electron flow and preventing overoxidation of PSI [94,95]. Although MF and DF1 started from a higher baseline, the S-deficit severely affected their re-reduction capability. The slow phase is more sensitive to stress [89,96], and it has been determined that the slow phase decreases progressively with stress intensity [97] as a result of a decreased rate of electron donation from PSII [98], a disconnection between PSII and PSI [99], or inhibition of PSI acceptor side [100].

The likely explanation is that older fronds require higher sulfur input to support their large pool of photosynthetic proteins. The S-deficit slows down the capacity to recycle electrons back to P700^+^. Since maternal fronds supply their daughters with nutrients, depending on the initial level of S storage, they cannot fully maintain their normal redox pool or turnover when one of the essential macronutrients is deficient [101]. Nutrient deficiency affects both the structural and functional performance of PSII photochemistry [102,103], and many papers show that chlorophyll fluorescence parameters could be relevant indicators of macronutrient or micronutrient deficiency [93,103,104,105,106,107].

However, GDF’s inherently lower oxidation and re-reduction rates do not decline under the S-deficit. They have a smaller PSI apparatus and a slower electron supply from PSII, as seen from the slow influx of electrons to reduce P700^+^. This suggests slower turnover and reflects lower photosynthetic capacity (rETR was lower). Nevertheless, their apparent resilience to S-deficit may have originated from lower metabolic S demands, preferential nutrient allocation from the mother frond until they mature, and/or protective or compensatory mechanisms that preserve PSI function despite S deficiency.

### 3.6. Chlorophyll Fluorescence—JIP Test

Under optimal conditions, older maternal fronds generally had higher performance indices (PI_ABS_, PI_TOT_, SFI_ABS_), again indicating a mature and stable photosynthetic apparatus. In contrast, younger fronds (DF2 and especially GDF) featured larger functional antenna sizes but exhibited lower efficiency of downstream electron transport, reflected in QA¯ accumulation due to incomplete transfer to the PQ pool. Although they capture light efficiently, these younger fronds still have an underdeveloped capacity to move electrons past PSII. So, to avoid over-reduction and photoinhibition, they dissipate excess energy as an essential protective strategy while their electron transport chains mature. Thus, as in Bielczynski et al. [82], prompt fluorescence measurements showed a clear ontogenetic trend: older fronds relied on well-optimized reaction centers and electron flow, whereas younger fronds showed comparatively higher photoprotective mechanisms.

Each age category responded differently when exposed to S deficiency, highlighting age × S-deficit interaction.

In MFs, the efficiency of absorbed light energy used in photochemistry within PSII remained unaffected, as did the efficiency of OEC. Slower electron flow from the PQ pool to reduce P700^+^ resulted from the accumulation of PQH_2_, signaling a bottleneck (as seen in the MR signal) [80,108]. Tikhonov [109] showed that the first step of PQH_2_ oxidation limits the rate of intersystem electron transport. Furthermore, Carstensen et al. [110] determined that phosphorus deficiency restricts downstream PQH_2_ oxidation, leading to PQH_2_ accumulation and restricting electron transport beyond the PQ pool. Although electrons were piling up in MF under the S-deficit, PSII was not shutting down or draining excessive energy as heat. However, this electron leakage may have led to the formation of reactive oxygen species that could cause damage. Nevertheless, fronds seemed to tolerate this partial over-reduction without immediate damage. Still, the overall performance was compromised due to a slowed electron transport rate despite no change in PSII activity.

All JIP test parameters indicated that sulfur deficiency severely affected the oldest daughters’ fronds (DF1). Because of weaker energetic connectivity or cooperation among PSII units, which led to a less efficient energy transfer, DF1 was the only frond with an imbalance of electrons arriving and leaving PSII RCs, either because of slower oxygen-evolving complex activity or faster electron transfer from the acceptor side [111]. A positive K-band is mainly associated with donor-side limitations, specifically partial inactivation or reduced efficiency of the water-splitting complex [111,112]. K-band was previously observed in plants affected by heat [113] or drought stress [114], but also in the early phases of leaf development [90] and during nutrient deficiencies [103,104].

According to the results, OEC was not supplying electrons at a sufficient rate. Thus, in combination with the downstream bottleneck, it created an overall imbalance in electron flow through PSII. As already observed from the MR_820_ signal, further downstream, S deficiency disrupted electron flow through PSI, reducing the PSI end electron acceptors pool and inhibiting PSI activity. Even though DF1 accelerated turnover to the terminal acceptors (faster half-time), it could not compensate for the reduced pool size of NADP^+^ [91,112]. This caused a bottleneck in the electron flow. With PSI jammed, electrons had nowhere to go because of the increased energy dissipation ratio of electrons passing through QA and QB. Therefore, QA¯ and PQH_2_ accumulated [115]. Without re-oxidized QA, PSII reaction centers became non-reducing. This over-reduction of the PQ pool slowed intersystem electron transport and promoted leakage of electrons, causing reactive oxygen species formation and potential photoinhibition, which could have affected the breakdown of the water-splitting complex. Therefore, in DF1, a carrier pool on the PSI acceptor side is a bottleneck under the S-deficit with severe consequences upstream.

In contrast to DF1, the youngest granddaughter frond (GDF) under S-deficit had decreased PSII antenna size, but steady trapped energy flux indicated that this smaller antenna still efficiently feeds the RCs. GDFs showed improved PSII unit cooperativity and used absorbed energy more efficiently in photochemistry, with less dissipation (higher photochemical vs. non-photochemical proportion). Because QA¯ quickly reoxidized, PSII was not over-reducing it. The results indicated some QA¯ accumulation later, suggesting that downstream steps were not perfectly keeping up. However, the PQ pool was balanced, and PSI dynamics were unchanged. Thus, the total electron flux through a relatively small PSI pool may be met without overwhelming the limited amount of Fe-S proteins [37].

Furthermore, ΔW_IP_ showed biphasic kinetics in GDF, with, at first, electrons going to smaller but less accessible and faster-filling PSI end acceptors, e.g., Fe-S proteins, ferredoxin pool, or possibly cyclic electron flow components, then transitioning to larger or more easily accessible PSI acceptors (NADP^+^), which take longer to reduce. Although the flux of electrons through the intersystem increased, not all were likely to reach NADP^+^ or did so less efficiently (δR_0_ decreased), thus suggesting that some electrons must have been diverted to alternative sinks, potentially to pseudo-cyclic flow (Mehler reaction) or cyclic electron flow (CEF). However, since more were coming, the net did not change (ψR_0_ and φR_0_), and since the net flux did not drop, the CBB cycle presumably continued at an adequate rate.

In CEF, electrons from ferredoxin are recycled into the PQ pool via the PGR5/PGRL1 complex [116,117] or NDH complex [118] without further reducing QA while driving the proton pump and ATP production [119], supporting increased starch synthesis. Another possible pathway for diverting electrons was chlororespiration, where NDH transfers electrons from ferredoxin to the PQ pool, while PTOX oxidizes excess PQH_2_ by transferring electrons to O_2_ and forming water [120,121,122]. This would reduce the need for NPQ, which is consistent with the observed reduction in dissipation.

There was no evidence of OEC impairment in the GDFs, which appeared to be fully functional. However, the net O_2_ evolution rate dropped significantly under the S-deficit. If OEC worked perfectly and followed the speed of linear electron flow, but less O_2_ was observed, O_2_ must have been used or consumed before it exited the tissue. Two possible major routes of O_2_ uptake include the Mehler reaction and chlororespiration.

In the Mehler reaction, electrons from PSI reduce O_2_ to superoxide and further to water [123]. However, NADPH is used to detoxify the generated ROS. But was there sufficient NADPH to regenerate ascorbate and glutathione in this reaction? With lower overall anabolic demand (dark respiration and growth were suppressed due to a metabolic push to store carbon, so NADPH was not utilized in fatty acid synthesis), GDF could still have sufficient NADPH for the functioning of the CBB cycle and ROS detoxification. Additionally, even if PSII was more efficient, total photon absorption was reduced, thereby reducing the electrons generated by water splitting, which could have contributed to the reduction in net O_2_ evolution we measured. Higher efficiency per RC does not necessarily mean a higher water-splitting rate, but fewer photons absorbed means fewer electrons were liberated from water.

Therefore, Mehler’s reaction, chlororespiration, and reduced light absorption could have reduced net O_2_ evolution in GDFs. At the same time, Mehler’s reaction could have served as an alternative electron sink, CEF for additional ATP, and chlororespiration to balance the PQ pool and consume O_2_. With all these active reactions, GDF under S-deficit avoids bottlenecks and stabilizes the PSI redox dynamic, thereby reducing the risk of photoinhibition under stress. Finally, unlike in older fronds, increased PI_ABS_ and PI_TOT_ actually emphasize improved light utilization in these fronds.

## 4. Materials and Methods

### 4.1. Plant Material and Experimental Setup

*Spirodela polyrhiza* (L.) Schleiden (RDSC Clone ID 5634) was pre-cultivated in Steinberg nutrient solution [124] under a 16/8 h light/dark photoperiod, with light intensity (PPFD) of 120 μmol s^−1^ m^−2^ (TLD and CWL 36W; Philips, Amsterdam, The Netherlands) and a temperature of 25 ± 1 °C. The nutrient solution was changed weekly during the pre-cultivation period, maintaining a constant doubling rate of approximately 2.5 days to ensure reproducible results [125].

For the experiment, pre-cultivated young colonies were inoculated into 350 mL Erlenmeyer flasks containing standard Steinberg medium (for control conditions). For sulfur-deficient conditions (S-deficit), fronds were inoculated into a modified Steinberg medium in which MgSO_4_ was replaced with MgCl_2_ at equimolar concentrations. Plants were cultivated for 6 days under the same conditions as described above. All further measurements were done at the onset of the light period on the oldest mother fronds (MF), first daughter fronds (DF1), second daughter fronds (DF2), and the youngest granddaughter fronds (GDF); for all analysis and observations, fronds were drawn from separate Erlenmeyer flasks.

### 4.2. Transmission Electron Microscopy (TEM)

*S. polyrhiza* frond cut-offs, varying in age and cultivated under optimal conditions, were fixated for 60 min in 1% glutaraldehyde in 50 mM of cacodylate buffer (pH 7.2). Samples were rinsed three times for 10 min with cacodylate buffer and then fixed in 50 mM of cacodylate buffer containing 1% OsO4 for 8–12 h at 4 °C in the dark.

The samples were then subjected to gradual dehydration, with increasing ethanol concentrations, and finally treated with propylene oxide. Spurr’s propylene oxide and epoxy resin mixture was used to embed dehydrated specimens in three infiltration phases for 2–3 h (2:1, 1:1, 1:2), gently agitating the vials at room temperature. Next, the specimens were immersed in a fresh Spurr’s mixture for 4–5 h at 40 °C, replaced with a new resin for 3–4 h, and subsequently allowed to polymerize at 65 °C for 48 h. Ultra-thin (80 nm) sections for TEM were cut with a Leica EM UC7 ultramicrotome (Leica Microsystems, Vienna, Austria). The sections were post-stained with 0.02 M of lead citrate containing 0.16 M of sodium hydroxide (during 3 min) and 2% uranyl acetate (during 10 min) and analyzed using a Zeiss Libra 120 Plus TEM (Carl Zeiss AG, Oberkochen, Germany). Micrographs were photographed using an XF416 4k camera (Tietz Video and Image Processing Systems GmbH, Gauting, Germany).

Since the embedded tissue sections were not cut using an isotropic, uniform, and random protocol, and since chloroplasts are light-oriented, creating a 2D profile biased for measurements, the one parameter whose visible cross-section does not depend on orientation due to its nearly spherical geometry and can be reliably measured was the diameter of the plastoglobuli. Therefore, to avoid potentially deceptive orientation-dependent parameters, a quantitative morphometric analysis of plastoglobuli was conducted on the TEM micrographs. Using ImageJ Version 1.54p, a total of 37 chloroplast sections were measured (total plastoglobuli n = 100) and statistically analyzed.

### 4.3. Pigment, Starch, and Total Anthocyanins Analysis

Pigments (Chlorophyll *a+b*) were extracted from 0.1 g of fresh weight with pure acetone overnight (24 h, −20 °C) and centrifuged at the highest speed for 10 min at 4 °C. Absorbances were measured at 470, 644.8, and 661.6 nm using a spectrophotometer (Specord 40, Analitik Jena, Jena, Germany). Chlorophyll content per dry weight was calculated according to [126].

For starch content, 0.2 g of fresh weight were homogenized in 4 mL of 18% (*w*/*v*) HCl, shaken for 60 min at 4 °C, and centrifuged for 20 min at 5000× *g*. Aliquots of the diluted supernatant were mixed with an equal volume of Lugol’s solution (0.5% KI, 0.25% I_2_ in water), and absorbance was measured at 530 and 605 nm using a spectrophotometer (Specord 40, Analytik Jenna). Starch content per dry weight was calculated according to [127].

Total anthocyanins were extracted according to Manicelli et al. [128] and Neff and Chory [129] from 0.1 g of fresh weight in 0.6 mL of methanol with 1% HCl overnight (24 h, −20 °C, dark), then diluted with 0.4 mL of Mili-Q H_2_O and mixed with an equal volume of chloroform. The mixture was vortexed and centrifuged at the highest speed for 10 min (4 °C). Aliquots of the supernatant were combined with equal amounts of 60% methanol in 1% HCl: 40% Mili-Q H_2_O, and absorbance was measured at 530 and 657 nm using a spectrophotometer (Specord 40, Analitik Jenna). Relative amounts of anthocyanins per sample were corrected for chlorophyll content by subtracting one-third of A_657_ from A_530_ and expressed as total anthocyanins per dry weight in cyanidin-3-glucoside equivalents (CGE) using its molecular weight (449.2 g mol^−1^) and molar extinction coefficient of 26,900 L mol^−1^ cm^−1^ [130].

### 4.4. Oxygen Production and Consumption Measurements

Oxygen evolution was measured at 25 °C using a Clark-type oxygen electrode in a Chlorolab2^+^ system (Hansatech Instruments Ltd., Norfolk, UK). Duckweeds primarily acquire carbon from gaseous CO_2_ in the air, especially in carbonate-free media in laboratory conditions. However, these water plants take up the majority of carbon in aqueous inorganic form [131]. Therefore, to ensure precise measurements of O_2_ evolution rates and provide a constant saturating CO_2_ concentration, a 0.1 M bicarbonate buffer (15 mL 0.1 M K_2_CO_3_, 85 mL 0.1 M NaHCO_3_, pH 8.9) was used [132]. Fronds were dark adapted, and then, with continuous stirring, changes in oxygen concentration were recorded at 2-min intervals under dark conditions and at photosynthetically active radiation (PAR) intensities of 7, 35, 75, 150, 300, 400, 600, and 800 μmol m^−2^ s^−1^ for light response curve. Net O_2_ exchange rates were derived by determining the slope of O_2_ evolution curves versus time at every light level.

This net oxygen production represents the combined outcome of several processes within the buffer-frond system: gross O_2_ production at Photosystem II (water splitting), Rubisco oxygenation, mitochondrial respiration, photorespiration (if present), and any other metabolic reactions, such as the Mehler reaction and nitrate reduction. Change in O_2_ evolution rate was plotted versus light intensity, and the point where this line crosses zero change in O_2_ was considered as the light compensation point of net photosynthesis (LCP). Oxygen consumption in the dark approximates mitochondrial respiration (dark respiration, DR). Both oxygen production and uptake rates reflect key metabolic rates. Each measurement was performed in triplicate, and rates were normalized to plant sample dry weight (μmol O_2_ g^−1^ DW h^−1^).

### 4.5. Rapid Light Curve

Chlorophyll fluorescence rapid light curves (RLCs) were measured using the Handy PEA+ (Hansatech Instruments Ltd., Norfolk, UK) with a predefined protocol. The protocol began with an initial quasi-dark period of up to 10 s to allow rapid primary electron acceptor (QA) reoxidation without substantial relaxation of the non-photochemical quenching [133]. The quasi-dark period was followed by a saturating pulse, which was followed by a short-stepped sequence (10 s) of actinic light, incrementally increasing intensity from 10 μmol m^−2^ s^−1^ to 1750 μmol m^−2^ s^−1^, each followed by a saturating pulse. The collected raw data of F (or Fs) and Fmʹ were used to calculate ΦPSII and plotted against actinic light intensity. The RLC model was fitted to the empirical light response model proposed by Platt et al. [134]: *P(E)* = *P_s_* [1 − exp(−*αE*/*P_s_*)] exp(−*βE*/*P_s_*), where *E* is photon irradiance, *α* is the initial light use efficiency, *β* is the photoinhibition coefficient, *P* is measured (r)ETR at particular irradiance (*ΦPSII* × *E* × *PFDa* × *Fraction PSII*), and *Ps* is the maximum photosynthetic output the sample could sustain if there were no photoinhibition. The default values of coefficients *PFDa* = 0.84 (for leaf absorptance) and *Fraction PSII* = 0.5 (as a fraction of absorbed energy delivered to PSII) were used in model estimation. Parameter optimization was performed in Excel using the GRG nonlinear method (Solver add-in), minimizing the sum of square residuals between observed and modelled rETR (SSE = Σ(P_obs_ − P_model_)^2^), with *α*, *β*, and *Ps* constrained to positive values in a Light Curve Template provided by Hansatech Instruments Ltd., UK. The best-fit curve for interpreting relative electron transport rate (rETR) versus actinic light intensity was based on the model when the coefficient of determination exceeded 0.95 and residuals showed no systematic trend. The obtained rETR is only proportional to the actual flux, hence, “relative” electron transport rate, and is labeled in arbitrary units (a.u.) for comparison.

From RLC, three distinct regions were identified: the initial slope of the linear phase at the sub-saturating light region (α, electron/photon), a coefficient indicating downregulation or photoinhibition during higher light steps (β), and rETR_ma_, estimated maximum electron transport rate. Ek (μmol m^−2^ s^−1^), the light-saturating parameter indicating the onset of photosynthetic saturation, was also calculated [74,87]. All RLC measurements were repeated 10 times for each frond age and treatment.

### 4.6. Prompt Fluorescence and MR_820_

Light-induced chlorophyll fluorescence and modulated reflection at 820 nm were recorded using a multifunctional plant efficiency analyzer (MPEA-1, Hansatech Instruments Ltd., Norfolk, UK). For details on the underlying measurement principles, see Strasser et al. [80] and Kalaji et al. [81]. Fronds of *S. polyrhiza* were dark-adapted for at least 20 min before measurements.

For the basic one-pulse protocol, the photosynthetic photon flux density (PPFD) was 3000 μmol photons m^−2^s^−1^, and the duration of the actinic flash at 625 nm was 1 s. Chlorophyll *a* fluorescence and modulated reflection were simultaneously recorded during the illumination period.

The three-pulse protocol was used to estimate the amount of oxidizable P700 and plastocyanin (PC) as maximum oxidizable/reducible amplitude (ΔMR_max_) [89,135]. For the initial measurements, the samples were illuminated with actinic red light (625 nm, 3000 μmol photons m^−2^ s^−1^) for 1 s. This was followed by a 60 s pre-acquisition period in darkness, establishing a stable background or redox equilibrium. The samples were then illuminated with far-red light (735 nm) at 50% of its maximum intensity for 10 s to reset the electron transport chain to an oxidized state, followed by another 60 s pre-acquisition period to stabilize at the new baseline. The third pulse of actinic red light (625 nm, 3000 μmol photons m^−2^ s^−1^, 1 s) was applied to determine the amount of P700 and PC that can be re-oxidized from the new baseline state.

According to Strasser et al. [80], the first reliable value of the MR_820_ signal is at 0.7 ms (MR_0_). The redox state of PSI electron carriers was characterized based on the MR/MR_0_ curve with characteristic parameters: fast phase (descending part of the curve), describing PC and P700 oxidation by the initial light, its amplitude (ΔMR_fast_/MR_0_ = (MR_0_ − MR_min_)/MR_0_), and the initial rate of P700 oxidation (V_ox_) as the slope from 0.7 to 2 ms; slow phase (the increasing part of the curve), describing the re-reduction of PC and P700, its amplitude (ΔMR_slow_/MR_0_ = (MR_max_ − MR_min_)/MR_0_), and the initial rate of P700^+^ re-reduction (V_red_) as the slope from 40 to 100 ms; and previously mentioned maximum amplitude of the 820 nm reflection signal (ΔMR_max_).

Relative variable fluorescence was calculated to visualize the effect of S deficiency on the dynamics of chlorophyll-*a* fluorescence transients in *S. polyrhiza* fronds of different ages. The difference kinetics of relative variable fluorescence (ΔW_t_) induction curves, normalized by F_0_ and F_K_ (O–K phase), F_0_ and F_J_ (O–J phase), F_0_ and F_I_ (O–I phase), and F_I_ and F_P(M)_ (I–P phase), were calculated as ΔW_t_ = W_t(S def)_ − W_t(control)_. Next, the JIP test was used to analyze specific points in the chlorophyll fluorescence transients to characterize energy transfer from PSII to PSI [78,80,111]. Briefly, fluorescence data consisted of OJIP amplitudes (Fo, Fm, Fv, Fl, Fk, Fj, and Fi), timing, QA turnover number, and area (t(Fm), t(Fj), Am, Sm, Mo, Sm/t(Fm), dVG/dto, dV/dto), relative variable fluorescence (Vk, Vl, Vj, Vi), specific energy fluxes per reaction centers (ABS/RC, DIo/RC, TRo/RC, ETo/RC, REo/RC), phenomenological energy fluxes per excited cross-section (ABS/CS, DIo/CS, TRo/CS, ETo/CS, REo/CS), quantum yields and probabilities (ϕ(Po), ψ(o), ϕ(Eo), δ(Ro), ϕ(Ro)), and performance indices (γ(RC)/(1-γ(RC)), ϕ(Po)/(1-ϕ(Po)), ψ(Eo)/(1-ψ(Eo)), δRo/(1-δRo), PI(abs), PI(tot), SFI(abs)) to describe overall photosynthetic vitality. For detailed descriptions of the JIP test, see Stirbet and Govindjee [78], Tsimilli Michael [77], and Kalaji et al. [111,136], and for the definition of parameters, see the Abbreviation list.

### 4.7. Statistical Analysis

All statistical analysis was performed in XLSTAT Software (Data Analysis and Statistical Solution for Microsoft Excel, Addinsoft 1995–2025, Paris, France). Prior to analysis, the assumptions for normality and homogeneity of variance were checked using the Shapiro-Wilk test to assess whether residuals followed a normal distribution and Levene’s test to determine if variances were equal among groups. When the test assumptions were met, a two-way ANOVA was conducted to evaluate the effect of sulfur deficiency, frond age, and their interaction. When a statistically significant effect was found (*p* < 0.05), a post-hoc Tukey HSD test was used for pairwise comparison of differences among groups. Six replicates for each treatment level and frond age were used for biochemical measurements, three for oxygen evolution measurements, and 10 for RLC, prompt fluorescence, and modulated reflection measurements.

In addition to *p* values, the magnitude of these effects was quantified using the omega squared (ω^2^) statistic, which represents the proportion of the total variance in the response variable, accounting for error variance as a less biased estimator for smaller samples, and Cohen’s f statistics, which indicate standardized differences between means independently of sample size, with larger values representing a stronger effect [137].

A nonparametric Kruskal-Wallis test was performed for plastoglobuli size and oxygen evolution measurements to assess differences in distribution across treatment levels and frond ages. As a post hoc test, multiple pairwise comparisons were conducted using the Conover–Iman procedure with a Bonferroni-corrected significance level. Hedges *d* was used to estimate the size of the difference between treatment and control groups for the maximum rate of net O_2_ production and the dark respiration rate [138,139].

Curve fitting of RLC measurements was performed using the GRG nonlinear method, minimizing the sum of the squared estimated errors (SSE) using a Microsoft Excel^®^ template with a Solver add-in provided by Hansatech Instruments.

## 5. Conclusions

*Spirodela polyrhiza* fronds showed distinct age-related differences in chloroplast ultrastructure, metabolic activity, and photosynthetic regulation, which were further modulated by sulfur availability. Under optimal S conditions, each frond has its role in the coordinated strategy of the colony. When exposed to the S-deficit, each generation adapted uniquely. Older maternal fronds (MF) showed partial tolerance due to stored reserves, and although they maintained functional PSII, they accumulated reduced PQ pools that slowed electron flow beyond PSII. The first-generation daughter frond (DF1) had a robust photosynthetic apparatus and high baseline capacity but became the most vulnerable under sulfur deficit. DF1 exhibited the largest drop in photosynthetic indicators, as well as limitations in the water-splitting complex and reduced PSI end-acceptor capacity, resulting in donor- and acceptor-side bottlenecks in electron transport and potential photoinhibition. On the other hand, GDF maintained reduced light absorption per PSII reaction center, balanced PQ pools, and redox-stable PSI through several alternative electron pathways, preventing bottlenecks and avoiding over-reduction and photoinhibition while diverting more carbon into starch and anthocyanin production.

Overall, faced with nutrient deficiency, the *S. polyrhiza* colony changed its “fast growth” strategy to “survival” mode. Maternal plants invested their resources and prioritized the survival of the offspring at their own expense. At the same time, younger fronds temporarily reduced growth, adjusted photosynthetic electron flows, accumulated energy reserves, and potentially delayed abscission. Therefore, coordinated and age-specific adjustments in photosynthesis and metabolite accumulation efficiency provided *S. polyrhiza* with the flexibility to respond, thereby retaining the colony’s fitness and facilitating rapid recovery when sulfur becomes available again.

## Figures and Tables

**Table 1:** Summary of the plastoglobuli size (diameter, μm, mean ± SD) for each frond age of *S. polyrhiza* and the statistical grouping based on the Kruskal-Wallis test with multiple pairwise comparisons using Dunn’s procedure with a Bonferroni corrected significance level of 0.0083.

PG Diameter (μm)	n	Mean	Standard Deviation	Dunn’s Differences, *p*-Values			Grouping
				DF1	DF2	GDF	
MF	40	0.797	0.244	27.346, 0.003	43.875, <0.0001	62.661, <0.0001	A
DF1	13	0.287	0.071	0	16.529, 0.127	35.315, 0.000	B
DF2	16	0.134	0.057		0	18.786, 0.035	BC
GDF	31	0.078	0.028			0	C

## Data Availability

The authors will make the raw data supporting this article’s conclusions available upon request.

## Supplementary Materials

The following supporting information can be downloaded at https://www.mdpi.com/article/10.3390/plants14131907/s1, Table S1: Results of a two-way ANOVA for the effects of S-deficit (S), frond age (FA), and their interaction (S × FA) on chlorophyll a+b, starch, and total anthocyanin content in S. polyrhiza four frond colonies. R^2^ is the proportion of the total variation in the model, ω^2^ is the proportion of the variance explained by each factor, and Cohen’s f is the standardized size of that effect; Table S2: Results of a two-way ANOVA for the effects of S-deficit (S), frond age (FA), and their interaction (S × FA) on rETR_max_, α, β, and Ek parameters derived from RLC curves of *S. polyrhiza* four frond colonies. R^2^ is the proportion of the total variation in the model, ω^2^ is the variance explained by each factor, and Cohen’s f is the standardized effect size of that factor; Table S3: Results of a two-way ANOVA for the effects of S-deficit (S), frond age (FA), and their interaction (S × FA) on the redox state and electron transport dynamics of photosystem I (PSI) reaction center (P700) and PC during fast and slow phases: ΔMR_fast_/MR_0_, ΔMR_slow_/MR_0_, V_ox_, and V_red_ in S. polyrhiza four frond colonies. R^2^ is the proportion of the total variation in the model, ω^2^ is the proportion of variance explained by each factor, and Cohen’s f is the standardized effect size of that factor; Table S4: Results of a two-way ANOVA for the effects of S deficit (S), frond age (FA), and their interaction (S × FA) on selected photochemical and performance parameters obtained from chlorophyll a fluorescence transients using the JIP test. For each parameter, the overall model statistics (F7,72 with corresponding R2 and *p*-values), followed by the main effects of S, FA, and S × FA, with F (df), *p*-values, and the proportion of variance explained (ω^2^), as well as Cohen’s f as standardized difference between means for the magnitude of an effect are given; Figure S1: *Spirodela polyrhiza* in control complete nutrient Steinberg medium (a) and S deficit conditions (b) in a modified Steinberg medium where MgSO_4_ was replaced with MgCl_2_ at equimolar concentrations. The average surface area was calculated using ImageJ, where the image was converted to an HSB stack (with the threshold adjusted to H 35–90°, S > 60, and V > 60 to preserve the yellow-green color of the fronds). For control fronds, the approximate surface area was 43.7 cm^2^, while for S-deficient fronds it was approximately 29.3 cm2 (because in places fronds overlapped, the automatic watershed-segmentation was used to cut between these fronds, and when they were fully adhered, the algorithm merged them into a single object, therefore surface area has a 5 to 10% error). Comparing the total surface area of fronds, ≈ 33.4% reduced growth per surface area was observed in S-deficient conditions.

## Abbreviations

The following abbreviations are used in this manuscript:

ABS/CS_0_	absorbed photon flux per excited cross-section of PSII (or also apparent antenna size) at t = 0
ABS/RC	average absorbed photon flux per PSII reaction center
DI_0_/RC	the flux of energy dissipated per active RC
ET_0_/RC	electron transport flux from Q_A_^−^ to PQ per active PSII
TR_0_/RC	maximum trapped exciton flux per active PSII
RE_0_/RC	electron transport flux from Q_A_^−^ to final PSI acceptors per active PSII
S_m_	the normalized area between the OJIP curve and the line Fm (a proxy of the number of electron carriers per electron transport chain)—representing energy necessary for the closure of all reaction centers
N	the number indicating how many times QA is reduced while fluorescence reaches its maximal value (number of QA redox turnovers until Fm is reached)
CS	CS, a cross-section of PSII
V_L_	relative variable fluorescence at L-band
V_K_	relative variable fluorescence at 300 μs (K-step)
V_J_	relative variable fluorescence at 2 ms (J-step)
V_I_	relative variable fluorescence at 30 ms (I-step)
V_K_/V_J_	the ratio of variable fluorescence in time 0.3 ms to variable fluorescence in time 2 ms as an indicator of the PSII donor side limitation
ΔV/Δto	initial slope (in ms-1) of O-J (the rate of accumulation of closed reaction centers), a ratio expressing the rate of accumulation of closed reaction centers
F_0_	initial fluorescence value
F_m_	maximal fluorescence intensity
F_V_	variable chlorophyll fluorescence (Fm − Fo)
F_V_/F_0_	the ratio between the rate constants of photochemicaland nonphotochemical deactivation of excited Chl molecules
F_V_/F_M_	the maximum quantum efficiency of PSII under dark adaptation
φP_0_	the maximum quantum yield of primary PSII photochemistry
ψE_0_	the efficiency with which a PSII-trapped electron is transferred from QA to QB
φE_0_	the quantum yield of electron transport from Q_A_^−^ to PQ
ψR_0_	the efficiency with which a PSII trapped electron is transferred to final PSI acceptors
φR_0_	the quantum yield of electron transport from Q_A_^−^ to final PSI acceptors
δR_0_	the efficiency with which an electron from QB is transferred to the final PSI acceptors
φD_0_	quantum yield of energy dissipation in PSII antenna (maximum quantum yield of nonphotochemical deexcitation)
PI_ABS_	performance index (potential) for energy conservation from exciton to reduction of intersystem electron acceptors
PI_TOT_	performance index (potential) for energy conservation from exciton to reduction of PSI end acceptors; QA and QB, primary and secondary quinone electron acceptors
SFI_ABS_	PSII structure-function index
PQ	the pool of free plastoquinones
PQH_2_	Plastoquinols
PC	plastocyanins
PSI	Photosystem I
PSII	Photosystem II
OEC	oxygen-evolving complex
DF	daughter frond
MF	mother frond
GDF	granddaughter frond
MR_t_	modulated 820-nm reflection intensity at time t
Vox	the rate of P700 oxidation
Vred	the rate of P700 re-reduction
LCP	Light compensation point
ETRmax	maximum relative electron transport rate at saturating light
rETR	relative electron transport rate
α	the slope of the linear phase at sub-saturating light of the ETR vs. E curves
β	coefficient of downregulation/photoinhibition
Ek	the minimum saturating irradiance
RLC	Rapid Light Curve
