# Supplementary material for: Age-Specific Physiological Adjustments of Spirodela polyrhiza to Sulfur Deficiency
Source: Plants (Basel). 2025 Jun 20;14(13):1907. doi: 10.3390/plants14131907 (PMC12252187; doi:10.3390/plants14131907)
Supplement: Supplementary file 1 [file plants-14-01907-s001.zip › plants-3654148-supplementary.pdf]

plants

MDPI

Table S1. Results of a two-way ANOVA for the effects of S-deficit (S), frond age (FA), and their interaction (S × FA) on chlorophyll *a+b*, starch, and total anthocyanin content in *S. polyrhiza* four frond colonies. R^2 is the proportion of the total variation in the model, ω^2 is the proportion of the variance explained by each factor, and Cohen's f is the standardized size of that effect.

Copyright: © 2025 by the authors. Licensee MDPI, Basel, Switzerland. This article is an open access article distributed under the terms and conditions of the Creative Commons Attribution (CC BY) license (https://creativecommons.org/licenses/by/4.0/).

Parameter	Factor	F-value and df (p < 0.0001)	R^2 / ω^2	Cohen's f
Chlorophyll *a+b*	Model	$F_{7,40}$ = 716.8***	0.99 (R^2)	-
	S-deficit (S)	$F_{1,40}$ = 3503.4***	0.693	9.36
	Frond Age (FA)	$F_{3,40}$ = 177.6***	0.105	3.60
	S × FA	$F_{3,40}$ = 322.5***	0.193	4.90
Starch	Model	$F_{7,40}$ = 77.8***	0.92 (R^2)	-
	S-deficit (S)	$F_{1,40}$ = 267.2***	0.455	2.60
	Frond Age (FA)	$F_{3,40}$ = 60.2***	0.303	2.10
	S × FA	$F_{3,40}$ = 32.2***	0.160	1.60
Total anthocyanins	Model	$F_{7,40}$ = 1078.4***	0.99 (R^2)	-
	S-deficit (S)	$F_{1,40}$ = 3592.8***	0.473	9.50
	Frond Age (FA)	$F_{3,40}$ = 850.7***	0.336	8.00
	S × FA	$F_{3,40}$ = 468.0***	0.185	5.90

[1] ω^2 is a less biased estimator; in Cohen's f, the larger the effect size, the stronger the relationship between variables; *** p < 0.0001.

Table S2. Results of a two-way ANOVA for the effects of S-deficit (S), frond age (FA), and their interaction (S × FA) on $rETR_{max}$, α, β, and Ek parameters derived from RLC curves of *S. polyrhiza* four frond colonies. R^2 is the proportion of the total variation in the model, ω^2 is the variance explained by each factor, and Cohen's f is the standardized effect size of that factor.

Parameter	Factor	F-value and df (p < 0.0001)	R^2 / ω^2	Cohen's f
rETRmax	Model	$F_{7,72}$ = 130.4***	0.93 (R^2)	-
	S-deficit (S)	$F_{1,72}$ = 354.4***	0.359	2.22
	Frond Age (FA)	$F_{3,72}$ = 163.4***	0.494	2.61
	S × FA	$F7_{,72}$ = 22.6***	0.066	0.97
α	Model	$F_{1,72}$ = 150.4***	0.94 (R^2)	-

	S-deficit (S)	$F_{3,72} = 74.4^{***}$	0.065	1.02
	Frond Age (FA)	$F_{3,72} = 309.3^{***}$	0.822	3.59
	S × FA	$F_{3,72} = 16.7^{***}$	0.042	0.83
β	Model	$F_{7,72} = 61.9^{***}$	0.86 (R^2)	-
	S-deficit (S)	$F_{1,72} = 210.6^{***}$	0.414	1.71
	Frond Age (FA)	$F_{3,72} = 56.4^{***}$	0.328	1.53
	S × FA	$F_{3,72} = 17.8^{***}$	0.100	0.86
Ek	Model	$F_{7,72} = 77.6^{***}$	0.88 (R^2)	-
	S-deficit (S)	$F_{1,72} = 446.8^{***}$	0.723	2.49
	Frond Age (FA)	$F_{3,72} = 19.8^{***}$	0.091	0.91
	S × FA	$F_{3,72} = 12.4^{***}$	0.055	0.72

[1] ω^2 is a less biased estimator; in Cohen's f, the larger the effect size, the stronger the relationship between variables; *** $p < 0.0001$.

Table S3. Results of a two-way ANOVA for the effects of S-deficit (S), frond age (FA), and their interaction (S × FA) on the redox state and electron transport dynamics of photosystem I (PSI) reaction center (P700) and PC during fast and slow phases: $\Delta MR_{fast}/MR_0$, $\Delta MR_{slow}/MR_0$, V_{ox}, and V_{red} in *S. polyrhiza* four frond colonies. R^2 is the proportion of the total variation in the model, ω^2 is the proportion of variance explained by each factor, and Cohen's f is the standardized effect size of that factor.

Parameter	Factor	F-value and df	R^2 / ω^2	Cohen's f
$\Delta MR_{fast}/MR_0$	Model	$F_{7,72} = 95.9^{***}$	0.89 (R^2)	-
	S-deficit (S)	$F_{1,72} = 33.9^{***}$	0.044	0.69
	Frond Age (FA)	$F_{3,72} = 204.3^{***}$	0.821	2.94
	S × FA	$F_{3,72} = 8.03^{**}$	0.028	0.58
$\Delta MR_{slow}/MR_0$	Model	$F_{7,72} = 79.9^{***}$	0.88 (R^2)	-
	S-deficit (S)	$F_{3,72} = 64.0^{***}$	0.091	0.91
	Frond Age (FA)	$F_{3,72} = 160.1^{***}$	0.756	2.60
	S × FA	$F_{3,72} = 6.7^{**}$	0.027	0.53
V_{ox}	Model	$F_{7,72} = 17.6^{***}$	0.60 (R^2)	-
	S-deficit (S)	$F_{1,72} = 0.06^{ns}$	-0.004	0.05
	Frond Age (FA)	$F_{3,72} = 39.0^{***}$	0.588	1.29
	S × FA	$F_{3,72} = 1.8^{ns}$	0.012	0.28
V_{red}	Model	$F_{7,72} = 56.4^{***}$	0.83 (R^2)	-
	S-deficit (S)	$F_{1,72} = 100.5^{***}$	0.203	1.17
	Frond Age (FA)	$F_{3,72} = 88.5^{***}$	0.572	1.95
	S × FA	$F_{3,72} = 9.4^{***}$	0.054	0.63

[1] ω^2 is a less biased estimator; in Cohen's f, the larger the effect size, the stronger the relationship between variables; *** $p < 0.0001$; ** $p < 0.001$; ^{ns} $p > 0.05$.

Table S4. Results of a two-way ANOVA for the effects of S deficit (S), frond age (FA), and their interaction (S × FA) on selected photochemical and performance parameters obtained from chlorophyll *a* fluorescence transients using the JIP test. For each parameter, the overall model statistics ($F_{7,72}$ with corresponding R^2 and p-values), followed by the main effects of S, FA, and S × FA, with F (df), p-values, and the proportion of variance explained (ω^2), as well as Cohen's f as standardized difference between means for the magnitude of an effect are given.

	ANOVA model	S deficit (S)			Frond Age (FA)			S × FA		
	$F_{7,72}$ (R^2, p-value)	$F_{1,72}$ (p-value)	ω^2	Cohen's f	$F_{3,72}$ (Pr > F)	ω^2	Cohen's f	$F_{3,72}$ (Pr > F)	ω^2	Cohen's f
F_v/F_0	22.21 (68%, < 0.0001)	2.34 (0.130)	0.006	0.176	38.96 (< 0.0001)	0.496	0.908	12.14 (< 0.0001)	0.146	0.622
V_L	86.82 (89%, <0.0001)	8.64 (0.004)	0.013	0.366	190.43 (< 0.0001)	0.833	2.796	9.04 (< 0.0001)	0.035	0.610
V_K	100.88 (91%, < 0.0001)	21.95 (< 0.0001)	0.030	0.574	222.97 (< 0.0001)	0.852	3.025	4.70 (0.005)	0.014	0.440
V_J	32.24 (76%, < 0.0001)	2.03 (0.158)	0.003	0.161	49.69 (< 0.0001)	0.484	1.423	25.30 (< 0.0001)	0.243	1.020
V_I	37.76 (78%, < 0.0001)	113.12 (< 0.0001)	0.330	1.243	13.78 (< 0.0001)	0.112	0.749	36.90 (< 0.0001)	0.318	1.231
V_K/V_J	14.03 (57%, < 0.0001)	8.89 (0.004)	0.046	0.349	13.43 (< 0.0001)	0.216	0.743	16.35 (< 0.0001)	0.267	0.820
S_m	32.42 (76%, < 0.0001)	64.60 (< 0.0001)	0.211	0.941	24.65 (< 0.0001)	0.233	1.001	29.74 (< 0.0001)	0.287	1.106
$\Delta V/\Delta(t)_0$	83.18 (89%, < 0.0001)	0.170 (0.681)	-0.001	0.066	169.19 (< 0.0001)	0.765	2.631	25.53 (< 0.0001)	0.112	1.024
ABS/CS_0	9.22 (47%, < 0.0001)	12.31 (0.001)	0.082	0.410	5.07 (0.003)	0.088	0.456	12.36 (< 0.0001)	0.246	0.713
ABS/RC	45.46 (81%, < 0.0001)	0.40 (0.527)	-0.002	0.063	96.24 (< 0.0001)	0.728	1.988	9.80 (< 0.0001)	0.067	0.635
DI_0/RC	15.36 (60%, < 0.0001)	6.31 (0.014)	0.029	0.294	25.09 (< 0.0001)	0.398	1.015	8.65 (< 0.0001)	0.126	0.596
TR_0/RC	100.88 (91%, < 0.0001)	24.04 (0.014)	0.030	0.574	222.66 (< 0.0001)	0.852	3.025	4.71 (< 0.0001)	0.014	0.440
ET_0/RC	14.14 (58%, < 0.0001)	9.47 (0.003)	0.049	0.360	11.34 (< 0.0001)	0.179	0.683	18.50 (< 0.0001)	0.303	0.872
RE_0/RC	22.48 (68%, < 0.0001)	66.04 (< 0.0001)	0.281	0.951	5.34 (0.002)	0.056	0.468	25.11 (< 0.0001)	0.313	1.016
φ(Po)	13.71 (57%, < 0.0001)	7.50 (0.008)	0.038	0.320	20.07 (< 0.0001)	0.337	0.908	9.42 (< 0.0001)	0.149	0.622
ψ(Eo)	32.24 (76%, < 0.0001)	2.03 (0.158)	0.003	0.161	49.69 (< 0.0001)	0.484	1.423	25.30 (< 0.0001)	0.243	1.020
φ(Eo)	32.49 (76%, < 0.0001)	1.85 (0.178)	0.002	0.153	51.25 (< 0.0001)	0.497	1.446	24.33 (< 0.0001)	0.232	1.000
δ(Ro)	39.58 (79%, < 0.0001)	196.75 (< 0.0001)	0.553	1.634	22.84 (< 0.0001)	0.186	0.968	4.55 (0.006)	0.030	0.432
φ(Ro)	31.21 (75%, < 0.0001)	68.70 (< 0.0001)	0.231	0.969	17.38 (< 0.0001)	0.167	0.842	32.74 (< 0.0001)	0.326	1.160
ψ(Ro)	37.76 (78%, < 0.0001)	113.12 (< 0.0001)	0.330	1.243	13.78 (< 0.0001)	0.112	0.749	36.90 (< 0.0001)	0.318	1.231
φ(Do)	13.71 (57%, < 0.0001)	7.50 (0.008)	0.038	0.320	20.07 (< 0.0001)	0.337	0.908	9.42 (< 0.0001)	0.149	0.622
PI_{ABS}	66.97 (87%, < 0.0001)	7.90 (0.006)	0.014	0.341	124.37 (< 0.0001)	0.678	2.254	29.85 (< 0.0001)	0.159	1.108
PI_{TOT}	53.33 (84%, < 0.0001)	84.46 (< 0.0001)	0.188	1.079	57.96 (< 0.0001)	0.379	1.537	38.61 (< 0.0001)	0.252	1.260
SFI_{ABS}	58.15 (85%, < 0.0001)	0.14 (0.705)	-0.002	0.056	110.67 (< 0.0001)	0.680	2.127	25.50 (< 0.0001)	0.153	1.024

[1] ω^2 is a less biased estimator; in Cohen's f, the larger the effect size, the stronger the relationship between variables.

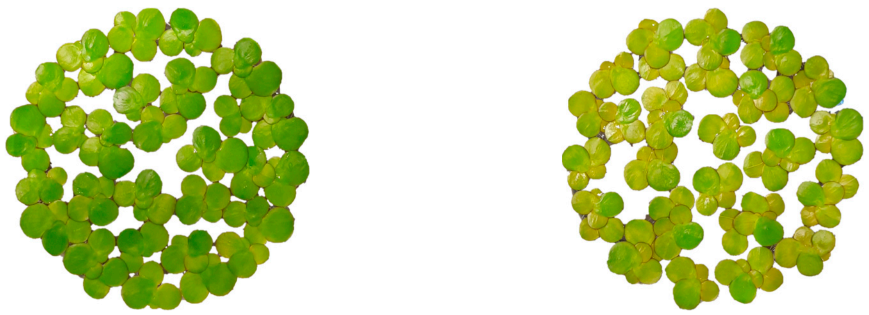

(**a**) (**b**)

Figure S1. *Spirodela polyrhiza* in control complete nutrient Steinberg medium (a) and S deficit conditions (b) in a modified Steinberg medium where $MgSO_4$ was replaced with $MgCl_2$ at equimolar concentrations. The average surface area was calculated using ImageJ, where the image was converted to an HSB stack (with the threshold adjusted to H 35–90°, S > 60, and V > 60 to preserve the yellow-green color of the fronds). For control fronds, the approximate surface area was 43.7 cm2, while for S-deficient fronds it was approximately 29.3 cm^2 (because in places fronds overlapped, the automatic watershed-segmentation was used to cut between these fronds, and when they were fully adhered, the algorithm merged them into a single object, therefore surface area has a 5 to 10 % error). Comparing the total surface area of fronds, $\approx$ 33.4 % reduced growth per surface area was observed in S-deficient conditions.
